# Aromatase and Dual Aromatase-Steroid Sulfatase Inhibitors from the Letrozole and Vorozole Templates

**DOI:** 10.1002/cmdc.201100145

**Published:** 2011-05-23

**Authors:** Paul M Wood, L W Lawrence Woo, Mark P Thomas, Mary F Mahon, Atul Purohit, Barry V L Potter

**Affiliations:** [a] Medicinal Chemistry, Department of Pharmacy and Pharmacology, University of BathClaverton Down, Bath BA2 7AY (UK), Fax: (+44) 1225-386-114; [b] X-Ray Crystallographic Suite, Department of Chemistry, University of BathClaverton Down, Bath, BA2 7AY (UK); [c] Division of Diabetes, Endocrinology & Metabolism, Imperial College London, Hammersmith HospitalLondon, W12 0NN (UK)

**Keywords:** aromatase, breast cancer, dual inhibitors, endocrine therapy, sulfatase

## Abstract

Concurrent inhibition of aromatase and steroid sulfatase (STS) may provide a more effective treatment for hormone-dependent breast cancer than monotherapy against individual enzymes, and several dual aromatase–sulfatase inhibitors (DASIs) have been reported. Three aromatase inhibitors with sub-nanomolar potency, better than the benchmark agent letrozole, were designed. To further explore the DASI concept, a new series of letrozole-derived sulfamates and a vorozole-based sulfamate were designed and biologically evaluated in JEG-3 cells to reveal structure–activity relationships. Amongst achiral and racemic compounds, 2-bromo-4-(2-(4-cyanophenyl)-2-(1*H*-1,2,4-triazol-1-yl)ethyl)phenyl sulfamate is the most potent DASI (aromatase: IC_50_=0.87 nm; STS: IC_50_=593 nm). The enantiomers of the phenolic precursor to this compound were separated by chiral HPLC and their absolute configuration determined by X-ray crystallography. Following conversion to their corresponding sulfamates, the *S*-(+)-enantiomer was found to inhibit aromatase and sulfatase most potently (aromatase: IC_50_=0.52 nm; STS: IC_50_=280 nm). The docking of each enantiomer and other ligands into the aromatase and sulfatase active sites was also investigated.

## Introduction

The growth and development of the most common form of breast malignancies, hormone-dependent breast cancer (HDBC), is promoted by the presence of oestrogenic steroids. Currently, the most widely used therapies for the treatment of this disease focus on blocking the action of these steroids, either by the use of selective, oestrogen receptor modulators, such as tamoxifen, or by inhibiting their biosynthesis through inhibition of the aromatase enzyme complex. Third-generation aromatase inhibitors (AIs), currently finding widespread application in the clinic, comprise the nonsteroidal compounds anastrozole and letrozole, and the steroidal exemestane.[1–3] Although these compounds were initially used in patients for whom tamoxifen therapy had failed, data from a number of clinical trials suggest that AIs provide a more effective first-line therapy against HDBC as a result of their superior efficacy and toxicology profile.[4–6] Of these AIs, there is evidence to suggest that letrozole is superior to anastrozole in suppressing oestrogen levels in breast tissue and plasma in patients with postmenopausal breast cancer.[7] It is unclear how this difference will translate to the clinic, however, the Femara versus Anastrozole Clinical Evaluation (FACE) trial should help to determine whether any differences in efficacy exist between these two AIs.[8]


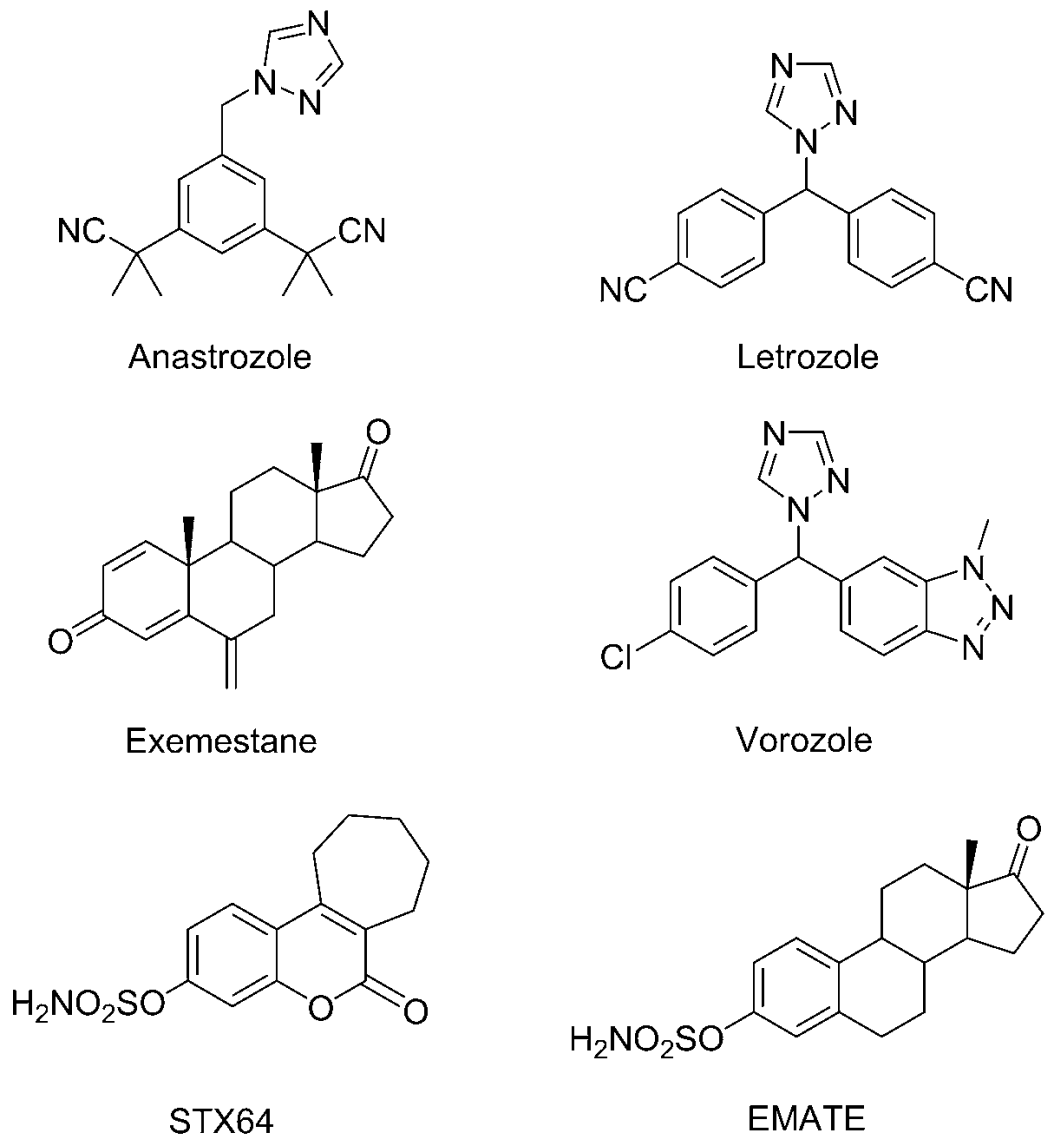


A promising new therapy for the treatment of HDBC has arisen from the development of inhibitors of steroid sulfatase (STS).[9] This enzyme is believed to be virtually ubiquitous throughout the body and is responsible for the conversion of alkyl and aryl steroid sulfates to their unconjugated and biologically active forms. Primarily, STS catalyses the conversion of oestrone sulfate, a biologically inactive steroid found at high levels in the plasma of postmenopausal women, to oestrone. In breast cancer tissue, it has been shown that ten times more oestrone originates from oestrone sulfate than from androstenedione.[10] In addition, STS controls the formation of dehydroepiandrosterone (DHEA) from DHEA-sulfate (DHEA-S). DHEA can be subsequently converted to androst-5-ene-3*β*,17*β*-diol, an androgen with oestrogenic properties capable of stimulating the growth of breast cancer cells in vitro[11] and inducing mammary tumours in vivo.[12] The pharmacophore for irreversible STS inhibition has been identified as a substituted phenol sulfamate ester, and a number of steroidal (e.g., oestrone-3-*O*-sulfamate, also known as EMATE) and nonsteroidal inhibitors (e.g., Irosustat, also known as STX64, BN83495) have been developed.[9, 13–14] Irosustat, discovered by our group, has been evaluated in a phase 1 clinical trial for the treatment of postmenopausal patients with metastatic breast cancer and has shown promising results.[15]

The advantages of a single chemical agent with the ability to interact with multiple biological targets have been recently highlighted.[16–18] A possible application of this concept for the treatment of HDBC would be the combination of the pharmacophores for both aromatase and STS inhibition into a single molecular entity. One approach to achieve this would be insertion of the pharmacophore for STS inhibition into an established AI, whilst maintaining the features necessary for aromatase inhibition. We previously reported three series of dual aromatase–sulfatase inhibitors (DASIs) based on different AIs: examples include, compounds **1** and **2** based on letrozole,[19–20] compound **3** based on YM511 (**4**),[21–24] and compound **5** based on anastrozole.[25] In a complementary approach, we also reported a series of DASIs obtained following introduction of the pharmacophore for aromatase inhibition into a biphenyl template primarily designed for STS inhibition (e.g, **6**).[26]


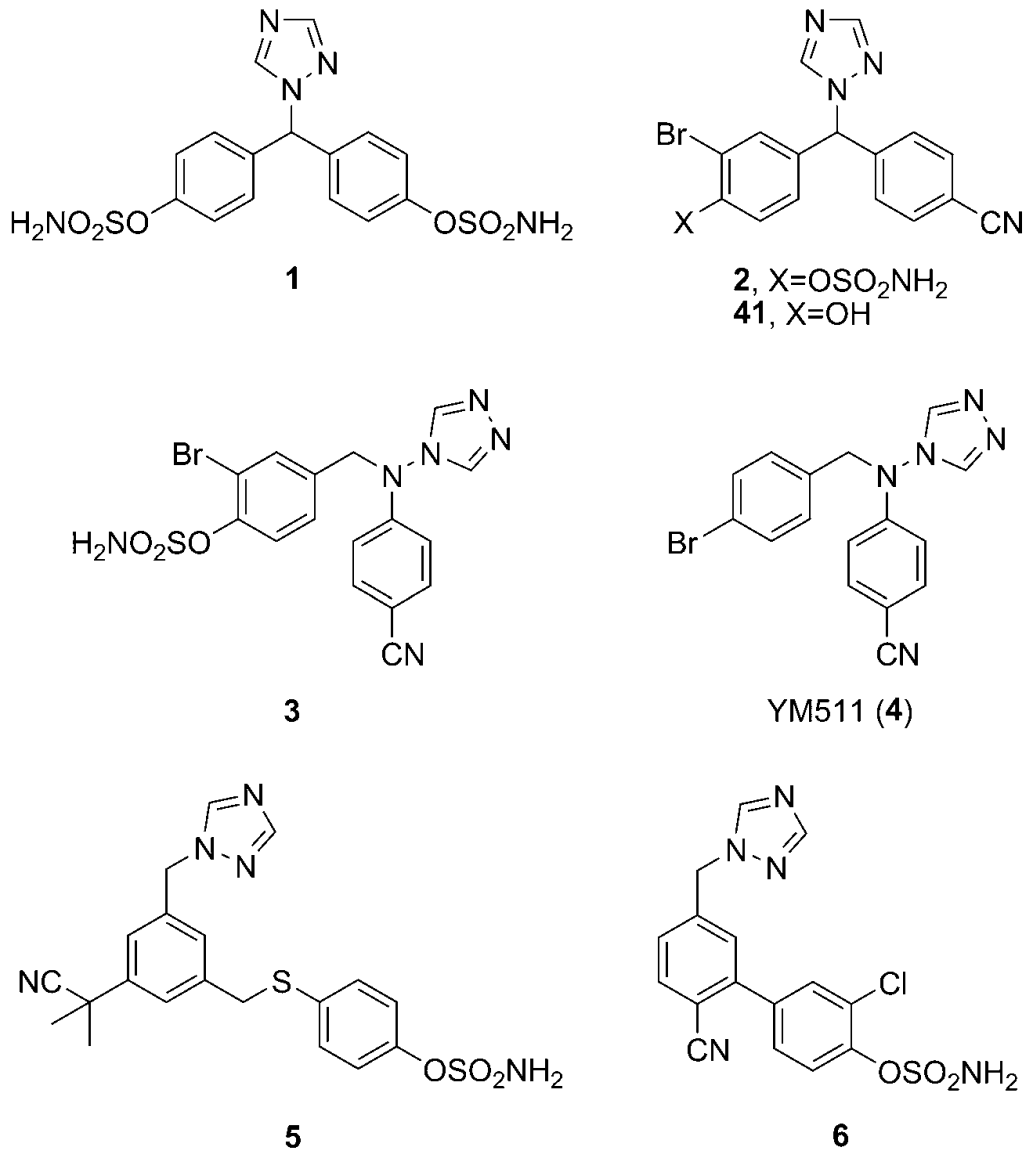


In preliminary work on the design of a prototype letrozole-based DASI, it was hoped that dual aromatase and sulfatase inhibition could be achieved by replacing both *para*-cyano groups present in letrozole with sulfamate groups, whilst retaining both the triazole and the diphenylmethane moieties necessary for potent aromatase inhibition.[19] A lead compound, *bis*-sulfamate **1** exhibited IC_50_ values of 3044 nm for aromatase and >10 μm for STS when evaluated in JEG-3 cells. Further iterations improved inhibition of both aromatase and sulfatase,[20] and the most potent AI identified was (±)-**2** in which only one of the *para*-cyano groups is replaced with a sulfamate group. Compound **2** inhibited aromatase and STS with IC_50_ values of 3 nm and 2600 nm, respectively. The enantiomers of **41**, the phenolic precursor of **2**, were separated by chiral HPLC and converted into their corresponding sulfamates;[27] the *R*-configuration provided the most potent aromatase inhibitor (*R*: 3.2 nm; *S*: 14.3 nm), whilst the *S*-configuration proved to be the best STS inhibitor (*S*: 553 nm; *R*: 4633 nm).[20]

Here, we report the further investigation of the structure–activity relationships (SAR) of letrozole-derived DASIs by evaluating the effect on inhibitory activity of increasing linker length between the triazole and the STS pharmacophore, and replacing the *para*-cyano-substituted ring with a *para*-chloro-substituted ring. The enantiomers of one compound were separated and their absolute configuration was determined by X-ray crystallography. We also report the synthesis and in vitro inhibitory activities of the first dual inhibitor derived from the third generation AI, vorozole.

## Results and Discussion

### Chemistry

**Scheme 1:**
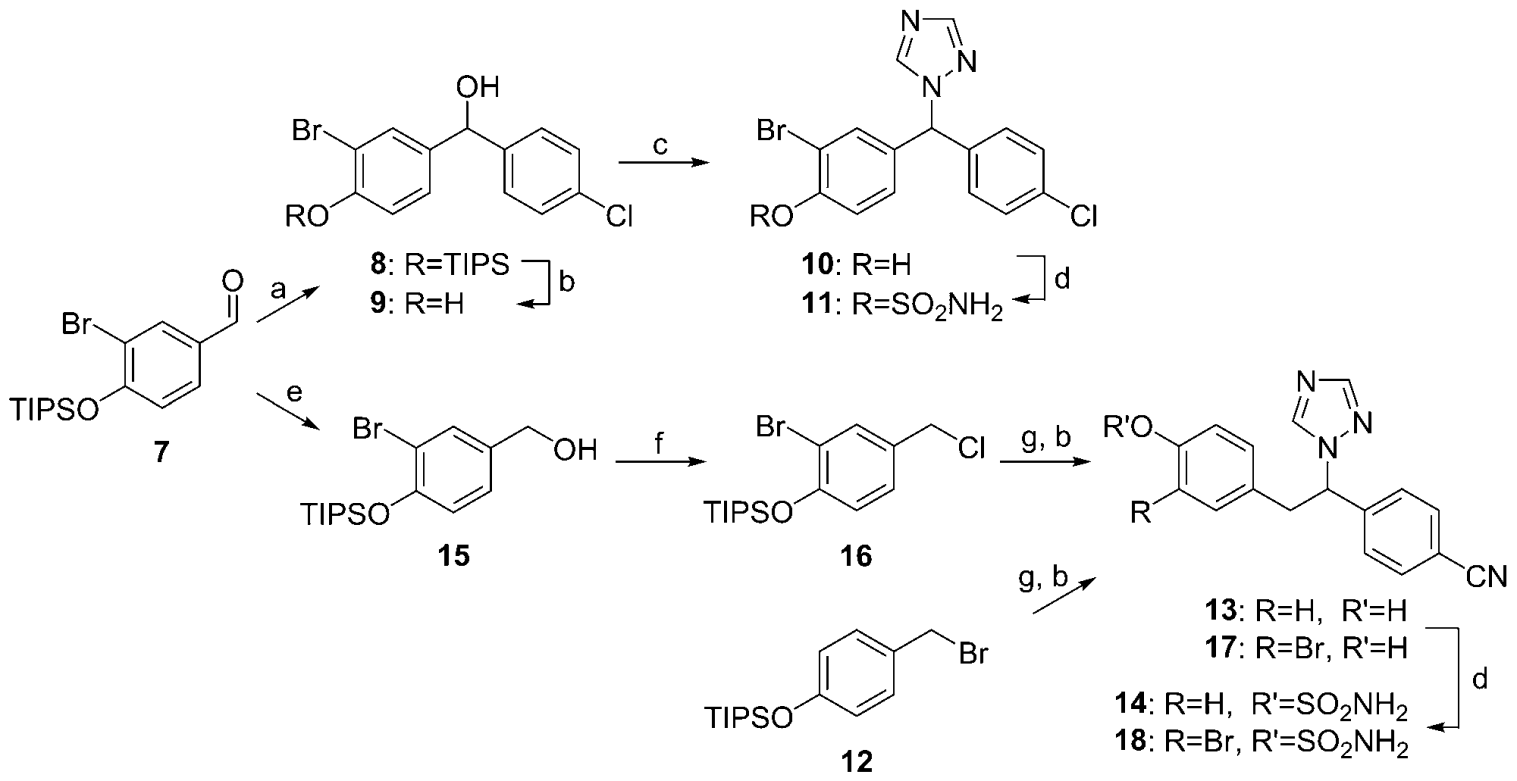
*Reagents and conditions*: a) 4-ClPhMgBr, THF, RT; b) TBAF, THF, RT; c) 1,2,4-Triazole, *p*-TsOH, toluene, reflux; d) H_2_NSO_2_Cl, DMA, RT; e) NaBH_4_, EtOH/H_2_O, RT; f) SOCl_2_, CH_2_Cl_2_, RT; g) *n*BuLi, 4-((1*H*-1,2,4-triazol-1-yl)methyl)benzonitrile, THF, −78 °C→RT.

**Scheme 2:**
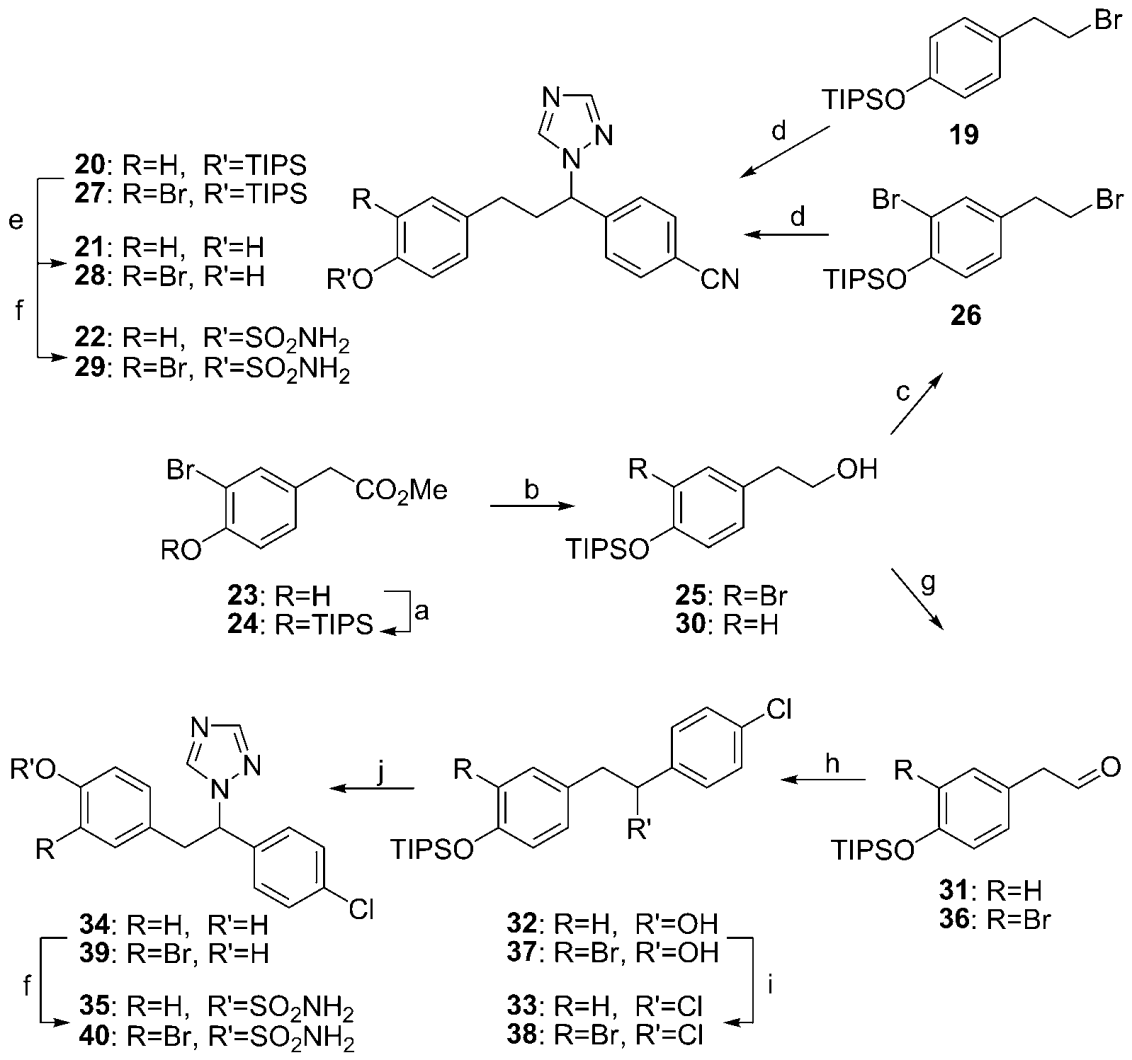
*Reagents and conditions*: a) Triisopropylsilyl chloride, imidazole, DMF, RT; b) LiBH_4_, B(OMe)_3_, Et_2_O, RT; c) Br_2_, PPh_3_, imidazole, Et_2_O/CH_3_CN, RT; d) *n*BuLi, 4-((1*H*-1,2,4-triazol-1-yl)methyl)benzonitrile, THF, −78 °C→RT; e) TBAF, THF, RT; f) H_2_NSO_2_Cl, DMA, RT; g) Dess–Martin periodinane, CH_2_Cl_2_, RT; h) 4-ClPhMgBr, THF, RT; i) SOCl_2_/DMF, CH_2_Cl_2_, RT; j) 1,2,4-Triazole, K_2_CO_3_, KI, acetone, 55 °C.

**Scheme 3:**
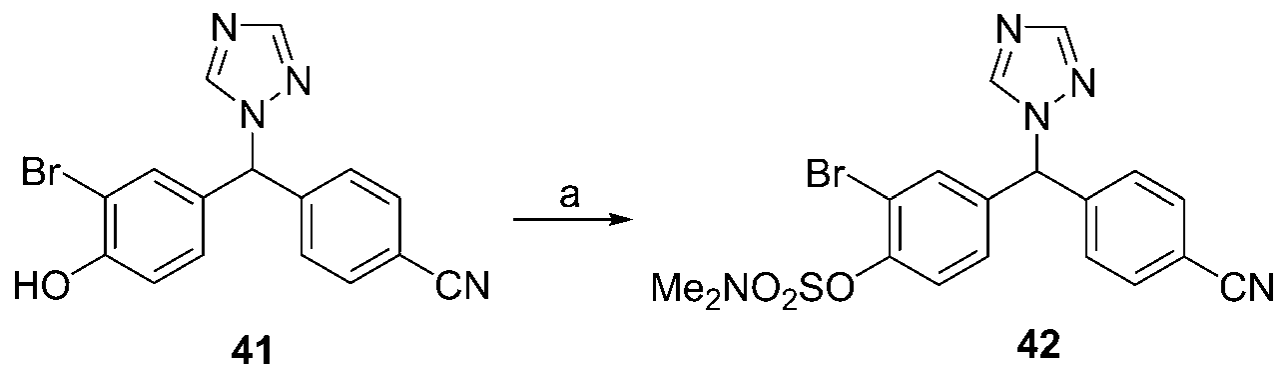
*Reagents and conditions*: a) *N*,*N*-Dimethylsulfamoyl chloride, DIPEA, reflux.

**Scheme 4:**
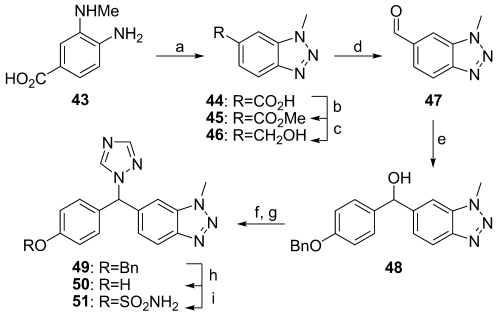
*Reagents and conditions*: a) NaNO_2_, 6 n HCl, H_2_O, 0 °C→RT; b) SOCl_2_, MeOH, reflux; c) LiAlH_4_, THF, RT; d) Trichloroisocyanuric acid, TEMPO, CH_2_Cl_2_, 0 °C, e) 4-Benzyloxybromobenzene, Mg, THF, RT; f) SOCl_2_, CH_2_Cl_2_, RT; g) 1,2,4-Triazole, KI, K_2_CO_3_, acetone, 60 °C; h) 10 % Pd/C, THF/MeOH, RT; i) H_2_NSO_2_Cl, DMA, RT.

All of the novel sulfamates and phenols described in this paper were prepared according to Schemes 1–4. The final compounds and intermediates were characterised by standard analytical methods and, in addition, the purity of the compounds tested in vitro was evaluated using HPLC. The reference AI, letrozole, was prepared by the alkylation of 1,2,4-triazole with α-bromomethyl-*p*-tolunitrile followed by reaction with 4-fluorobenzonitrile according to the procedure described by Bowman et al.[19, 28] The reference STS inhibitor, STX64, was synthesised according to the method of Woo et al.[29]

The synthetic route for the sulfamates **11**, **14** and **18** is shown in Scheme 1. Both sulfamates **11** and **18** were prepared from 3-bromo-4-(triisopropylsilyloxy)benzaldehyde (**7**)[20] and sulfamate **14** was prepared from (4-(bromomethyl)phenoxy)triisopropylsilane (**12**).[30] For **11**, treatment of aldehyde **7** with 4-chlorophenylmagnesium bromide followed by deprotection of the phenol afforded **9**. Displacement of the hydroxy group in **9** was achieved with 1,2,4-triazole in refluxing toluene to afford **10**, which was converted to sulfamate **11** by reaction with an excess of sulfamoyl chloride in *N,N*-dimethylacetamide (DMA) according to the conditions described by Okada et al.[31] For the synthesis of sulfamate **14**, it was envisaged that alkylation of *n*-butyllithium-deprotonated[32] 4-((1*H*-1,2,4-triazol-1-yl)methyl)benzonitrile[19] with (4-(chloromethyl)phenoxy)triisopropylsilane would provide a route to **13**; this reaction failed to provide the desired product. However, when the alkylating agent was switched to the more reactive **12**, the desired product could be obtained. Deprotection of the phenol was achieved using tetra-*n*-butylammonium fluoride and the product could be used without further purification. Phenol **13** was subsequently converted to sulfamate **14** using the conditions described above. Starting from aldehyde **7**, reduction with sodium borohydride and conversion of the resulting benzyl alcohol to the chloride gave compound **16**. This is a more reactive alkylating agent than its nonbrominated counterpart, and it was successfully used as the alkylating agent for the synthesis of sulfamate **18** according to the route described above.

The synthesis of sulfamates **22**, **29**, **35** and **40** is detailed in Scheme 2. Compound **22** was obtained in three steps from **19**, which was prepared according to Avery et al.[33] Reaction of **19** with 4-((1*H*-1,2,4-triazol-1-yl)methyl)benzonitrile as described above was followed by deprotection and sulfamoylation to furnish **22**. Sulfamate **29** was prepared in a similar manner using bromide **26**, which was itself prepared from methyl 2-(3-bromo-4-hydroxyphenyl)acetate **23**.[23] Following triisopropylsilyl (TIPS) protection of the phenolic hydroxy group in **23**, ester **24** was reduced with lithium borohydride and the resulting benzyl alcohol was converted to benzyl bromide **26**, and the synthesis of **29** was completed using the steps described above. The bottom part of Scheme 2 describes the route for the synthesis of sulfamates **35** and **40**. Sulfamate **35** was synthesised from **30**, which was prepared as described by Avery et al.[33] From alcohol **30**, Dess–Martin oxidation gave aldehyde **31**, which was reacted with 4-chlorophenylmagnesium bromide to give **32**. The alcohol was converted to chloride **33** with thionyl chloride, and this was reacted with 1,2,4-triazole in acetone with concomitant loss of the TIPS protecting group to give **34**. Finally, sulfamoylation as described above furnished **35**. Sulfamate **40** was prepared analogously from **23** following TIPS protection of the phenol and lithium borohydride reduction of ester **24**.

*N,N*-Dimethysulfamate **42** was successfully prepared by heating a mixture of 1-[(4-cyanophenyl)(3-bromo-4-hydroxyphenyl)methyl]-1*H*-[1,2,4]triazole[20] and *N,N*-dimethylsulfamoyl chloride in *N*,*N*-diisopropylethylamine (DIPEA) (Scheme 3).

Vorozole-derived sulfamate **51** was prepared from benzoic acid **43**, which was synthesised as described by Dener et al.[34] from 3-methoxy-4-nitrobenzoic acid (Scheme 4). Formation of the benzotriazole ring was achieved by treatment of **43** with a mixture of sodium nitrite and hydrochloric acid in water. Subsequent formation of methyl ester **45** and lithium borohydride reduction gave compound **46**. This approach to the synthesis of the benzotriazole ring ensures that the methyl group is placed on the correct nitrogen atom in the triazole ring. A more concise route to **45** was explored via the alkylation of methyl-1*H*-benzotriazole-5-carboxylate with methyl iodide in the presence of potassium carbonate but this gave a mixture of three regioisomers from which it was difficult to separate the individual benzotriazol-1-yl isomers. The oxidation of alcohol **46** with potassium permanganate in dichloromethane[35] gave aldehyde **47** in moderate yields. However, excellent yields of the aldehyde could be obtained by oxidation with the trichloroisocyanuric acid/catalytic 2,2,6,6-tetramethylpiperidinooxy (TEMPO) system reported by Giacomelli et al.[36] Aldehyde **47** was subsequently reacted with the Grignard reagent generated from 4-benzyloxybromobenzene to give alcohol **48**. This was converted to the corresponding chloride with thionyl chloride and quickly reacted with 1,2,4-triazole in the presence of potassium carbonate to give **49**. Deprotection of the phenol was achieved by catalytic hydrogenation with palladium on carbon to give **50**, and the formation of the corresponding sulfamate was achieved using the conditions described above to furnish **51**.

### Inhibition of aromatase and steroid sulfatase activity by sulfamoylated compounds

The in vitro inhibition of aromatase and STS activity by each sulfamate was measured in a preparation of an intact monolayer of JEG-3 cells. The results are reported as either IC_50_ values or as a percentage of inhibition at 10 μm, and are compared to the reference AI letrozole[19] and the reference STS inhibitor STX64[21] (Table 1). The biological activities of both **2** and **52** have been reported previously.[20]

**Table 1:** In vitro inhibition of the aromatase and STS activity in JEG-3 cells by letrozole, STX64, **52**, **2** and the sulfamates described herein

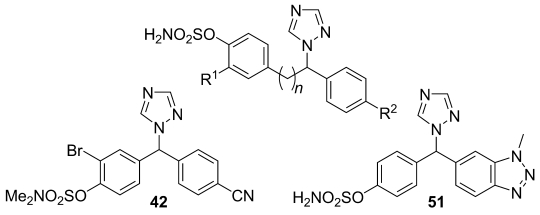					
Compd	R^1^	R^2^	*n*	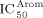 [nm]	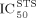 [nm]
Letrozole	–	–	–	0.89±0.13[a]	–
STX64	–	–	–	–	1.5±0.3[b]
**2**	Br	CN	0	3.0±0.1[c]	2600±100[c]
**11**	Br	Cl	0	2.5±0.7	1400±234
**14**	H	CN	1	2.8±0.2	3517±625
**18**	Br	CN	1	0.87±0.21	593±6
**22**	H	CN	2	0.22±0.01	>10 000
**29**	Br	CN	2	0.12±0.02	>10 000
**35**	H	Cl	1	39±10	2233±666
**40**	Br	Cl	1	1.4±1.1	180±26
**42**	–	–	–	23.04±2.92	>10 000
**51**	–	–	–	29±10	>10 000
**52**	H	CN	0	13±4[c]	21.5 %[c,d]

[a] Data taken from Wood et al.[19]

[b] Data taken from Woo et al.[21]

[c] Data taken from Wood et al.[20]

[d] Percent inhibition at 10 μm.

Mean IC_50_ values ±SD were determined from incubations carried out in triplicate in a minimum of two separate experiments.

All of the sulfamates tested in this series are potent inhibitors of aromatase with IC_50_ values ≤39 nm and, in addition, some compounds also exhibit moderate-to-potent STS inhibition. In this assay, two compounds, **22** and **29** (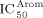
=0.22 and 0.12 nm, respectively) are more potent than the reference AI letrozole (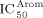
=0.89 nm).

Several compounds in this study contain a *para*-chloro-substituted phenyl ring rather than the *para*-cyano-substituted ring found in letrozole and letrozole-based DASI **2**. This substitution is present in compounds capable of potent aromatase inhibition. For instance, this moiety is present in the potent AI (±)-vorozole (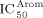
=2.59 nm),[37] and furthermore, a derivative of letrozole with both *para*-cyano groups replaced by *para*-chloro groups has been reported to inhibit aromatase activity with an EC_50_ value of 8.8 nm in a rat ovarian microsome assay.[38] For this series, comparison of the activities of the *para*-cyano-substituted compounds with their *para*-chloro-substituted counterparts reveals that, for the two sets of compounds **2**/**11** and **18**/**40**, dual inhibitory activity is retained following the switch in substitution (e.g., **2**: 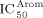
=3.0 nm, 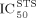
=2600 nm, vs **11**: 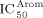
=2.5 nm, 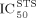
=1400 nm). However, for compounds **14** and **35**, replacement of the *para*-cyano group with a *para*-chloro group maintains STS inhibitory activity but is detrimental to aromatase activity (**14**: 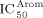
=2.8 nm, 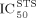
=3517 nm, vs **35**: 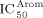
=39 nm, 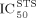
=2233 nm). The importance of the positioning of a hydrogen-bond acceptor (e.g., CN, NO_2_) in the molecule relative to the triazole/imidazole ring for potent aromatase inhibition has been extensively discussed in the literature.[39–40] Interestingly, in this series, replacement of the cyano group with the weaker hydrogen-bond accepting chloro substituent maintains good aromatase inhibitory activity, possibly due to a complex interaction between the hydrogen-bond donor in the active site, the halide and the π system of the connecting aromatic ring.[41]

The effect played by the linker between the aromatase and STS pharmacophores on dual inhibitory activities is illustrated by three series of compounds: 1) **52**, **14** and **22**; 2) **2**, **18** and **29**; 3) **11** and **40**. In each series, lengthening the linker (*n*) results in an increase in aromatase inhibition; this is illustrated by comparing compounds **2** (*n*=0; 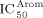
=3.0 nm), **18** (*n*=1; 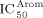
=0.87 nm), and **29** (*n*=2; 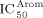
=0.12 nm). This correlates with the small increase in aromatase inhibition observed in the αω-diarylalkyltriazole series of compounds with inhibitory activities for 4-(3-(4-fluorophenyl)-1-(1*H*-1,2,4-triazol-1-yl)propyl)benzonitrile and linker extended 4-(4-(4-fluorophenyl)-1-(1*H*-1,2,4-triazol-1-yl)butyl)benzonitrile reported as 0.19 μm and 0.12 μm, respectively.[32] These finding are also in agreement with those in a YM511-derived DASI series, with activities for 4-(((4-cyanophenyl)(4*H*-1,2,4-triazol-4-yl)amino)methyl)phenyl sulfamate and 4-(2-((4-cyanophenyl)(4*H*-1,2,4-triazol-4-yl)amino)ethyl)phenyl sulfamate being 100 nm and 2.1 nm, respectively.[23] A small increase in linker length is beneficial for enhanced STS inhibition (**2**: 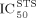
=2600 nm, vs **18**: 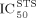
=593 nm), but further extension of the linker has a detrimental effect on inhibitory activity (**29**: 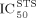 >10 000 nm). Extending the linker length was shown to be detrimental to STS activity in the YM511-derived DASI series, with a decrease in activity from 227 nm for 4-(((4-cyanophenyl)(4*H*-1,2,4-triazol-4-yl)amino)methyl)phenyl sulfamate to >10 000 nm for 4-(2-((4-cyanophenyl)(4*H*-1,2,4-triazol-4-yl)amino)ethyl)phenyl sulfamate, which has one extra methylene unit in the linker. This decrease in STS inhibition could be due to an increase in flexibility in the molecule as the linker is extended, resulting in less favourable binding of the compound in the active site.

As in our previous investigation into letrozole- and YM511-derived DASIs, derivatives containing a halogen positioned *ortho* to the sulfamate are better AIs than their nonhalogenated counterparts. This trend is exhibited in both the *para*-cyano- (**14**: 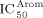
=2.8 nm vs **18**: 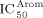
=0.9 nm) and *para*-chloro-substituted series (**35**: 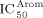
=39 nm vs **40**: 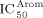
=1.4 nm). This and previous results suggest that the higher aromatase inhibition can be attributed to the increased lipophilicity conferred by the halogen.[20] Similarly, we previously discovered that the presence of a halogen group *ortho* to the sulfamate increases STS inhibitory activity, and this trend holds true in this series for both pairs of compounds **14**/**18** (**14**: 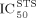
=3517 nm vs **18**: 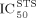
=593 nm) and **36**/**40**. The increase in STS inhibitory activity is reasoned to be caused by a lowering of the p*K*_a_ of the phenol, enhancing its leaving group ability. Presumably, the deleterious effect on activity caused by an increase in linker length for compounds **22** and **29** is too large for any halogen-induced increase in inhibition to be observed.

The *N,N*-dimethylsulfamate-containing compound **42** is a weaker AI than its corresponding demethylated counterpart **2**, despite the increase in lipophilicity conferred by dimethylation, which normally benefits aromatase inhibition. The weak STS inhibition exhibited by **42** in vitro is anticipated based on our previous work on *N,N*-dimethylated sulfamates.[13, 42] For example, despite the poor inhibitory activity against STS[43] exhibited by the *N,N*-dimethylated derivative of STX64 in vitro, this compound has been shown to almost completely inhibit mouse liver and skin STS activities 24 h after oral administration.[44] This suggests that *N,N*-demethylation may occur in vivo to provide a compound capable of STS inhibition, and it might also be the case that, although **42** is inactive in vitro, it may act as a prodrug of **2** in vivo. Further work is required to explore the potential in vivo conversion of compound **42** to **2**.

Compound **51** is the first reported example of a sulfamate-containing vorozole derivative. Vorozole is a third generation, aromatase-selective AI, that entered phase 3 clinical trials, but further development was discontinued when no improvement in median survival was obtained compared to megestrol acetate.[45] Nonetheless, we explored the feasibility of designing a DASI that is structurally related to vorozole. Incorporation of the STS inhibitory pharmacophore into the molecule was achieved by replacement of the *para*-chloro substituent attached to the phenyl ring in vorozole with a sulfamate group. Compound **51** exhibits good inhibitory activity against aromatase, although it is a weaker AI than the corresponding letrozole derivative **52** and, like **52**, also exhibits poor STS inhibition. However, based on previous observations, the introduction of appropriate substitutions onto the sulfamate-bearing ring in **51** would be expected to improve inhibitory activity against both enzymes, indicating the feasibility of a DASI based on the vorozole template.

### Inhibition of aromatase activity by parent phenols

The aromatase inhibition for the phenols described in this paper is tabulated in Table 2. The loss of the sulfamate group following irreversible inactivation of STS by a sulfamate-based DASI will result in the formation of the corresponding phenol. The quantity of phenol produced by this mechanism is limited in principle once all the STS activity has been inactivated.[46] However, degradation of the sulfamate following prolonged circulation in plasma might provide an additional route for the formation of the phenol. As these phenols still contain the pharmacophore for aromatase inhibition they have the potential to act as AIs in their own right.

**Table 2:** In vitro inhibition of the aromatase activity in JEG-3 cells by letrozole and the phenols described herein

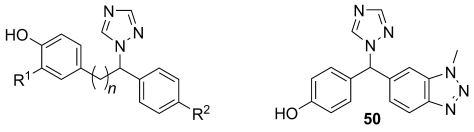				
Compd	R^1^	R^2^	*n*	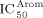 [nm]
Letrozole	–	–	–	0.89±0.13[a]
**10**	Br	Cl	0	2.3±0.8
**13**	H	CN	1	2.9±0.3
**17**	Br	CN	1	0.21±0.02
**21**	H	CN	2	0.16±0.02
**28**	Br	CN	2	0.02±0.01
**34**	H	Cl	1	80±5
**39**	Br	Cl	1	1.8±0.5
**50**	–	–	–	3.8±1.0

[a] Data taken from Wood et al.[19] Mean IC_50_ values ±SD are shown, determined from incubations in triplicate from two independent experiments.

The most potent phenol in this series against aromatase is **28** (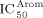
=0.02 nm) and three compounds, **17**, **21** and **28** (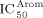
=0.21, 0.16, 0.02 nm, respectively) are more potent than the reference AI letrozole (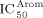
=0.89 nm) in this assay. With the exception of **34**, the phenols are either equipotent or slightly better inhibitors of aromatase compared to their corresponding sulfamates.

In common with the trends observed for their sulfamoylated counterparts, positioning a halogen *ortho* to the phenol results in an increase in aromatase inhibitory activity, as seen for example in compounds **13** and **17** (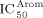
=2.9 nm vs 0.21 nm, respectively), and lengthening the linker is also beneficial for aromatase inhibition, as seen for example in compounds **13** and **21** (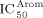
=2.9 nm vs 0.16 nm, respectively).

### Chiral HPLC and absolute structure determination

In order to enrich the SAR for letrozole-derived DASIs with their target proteins and to allow comparison with the inhibitory activities of the enantiomers of **2**, the activities of each enantiomer of **18**, one of the most promising DASIs in this current series, were determined. To avoid any complications arising from decomposition of the sulfamate during separation, resolution by chiral HPLC was performed with **17**, the parent phenol of the sulfamate, an approach previously used in the preparation of the enantiomers of **2**.[20]

The literature contains a number of reports on the resolution of AIs by chiral HPLC with a particular focus on imidazole-containing compounds: for example, fadrozole hydrochloride, which was separated with a Chiralcel OD column.[47] Using conditions similar to those we reported previously for the separation of phenol **43**, the enantiomers of phenol **17** were separated on a Chiralpak AD-H analytical column with methanol as the mobile phase (see Experimental Section for further details). The first enantiomer eluted from the column with a retention time of 3.80 min (**17 a**), whereas the second enantiomer eluted with a retention time of 8.2 min (**17 b**) giving greater peak separation than that previously obtained for **43**. This separation was subsequently scaled-up and successfully performed on a Chiralpak AD-H semi-prep column to separate 700 mg of the racemate with injections of 1.5–2.0 mL of a 20 mg mL^−1^ methanol solution of **17**. Conversion of **17 a** and **17 b** into their corresponding sulfamates was achieved with excess sulfamoyl chloride in DMA. We previously reported that the sulfamoylation step proceeds without loss of enantiomeric purity in the preparation of the enantiomers of **2**, **2 a** and **2 b**.[20] The optical rotation for each enantiomer of the phenol and corresponding sulfamate was measured (data given in the Experimental Section).

**Figure 1:**
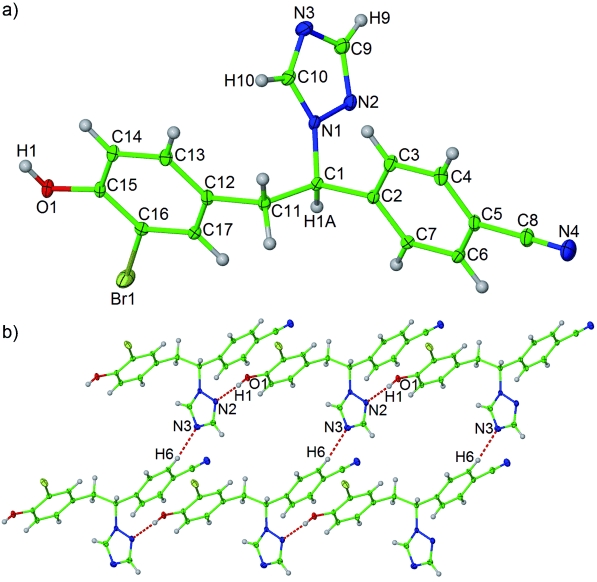
a) X-ray crystal structure of **17 a** (CCDC deposition code: 806541); ellipsoids are represented at 30 % probability. b) Portion of extended structure present in **17 a** illustrating the network of intermolecular hydrogen bonding.

Previously, in the absence of suitable crystals of **2 a**,**b** and **41 a**,**b** for X-ray analysis, the absolute configuration of each enantiomer had to be established using vibrational and electronic circular dichroism in conjunction with time-dependent density functional theory calculations of their predicted properties. Fortuitously, crystals suitable for X-ray analysis could be obtained from ethyl acetate solutions of both **17 a** and **17 b**, and the absolute configuration of each enantiomer was determined from the X-ray crystal structure of **17 a**.[48] The crystal structure obtained for **17 a** is shown in Figure 1, allowing the unambiguous elucidation of the absolute configuration of **17 a** as *R*-(−). Figure 1 further illustrates the sheets that stack along the *b* axis in the gross structure as a consequence of intermolecular hydrogen bonding between the phenolic hydrogen (H1) and N2 of a proximate triazole in the crystal: [H1–N2, 1.94 Å; O1⋅⋅⋅N2, 2.744 Å, O1–H1⋅⋅⋅N2, 174.8°]. The second C–H⋅⋅⋅O type interaction arises between H6 in one molecule and a triazole nitrogen (N3) from a lattice neighbour: [H6–N3, 2.34 Å; C6⋅⋅⋅N3, 3.29 Å; C6–H6⋅⋅⋅N3, 172.6°].

### Inhibitory activities of chiral sulfamates and their parent phenols

The difference in aromatase and STS inhibition exhibited by each enantiomer of **18** was evaluated following separation of the enantiomers of phenolic precursor **17** by chiral HPLC and conversion to their corresponding sulfamates. For comparison, the aromatase and STS inhibitory activities of each enantiomer of **18** and the aromatase inhibitory activities of the enantiomers of **17** are shown in Table 3 along with those previously obtained for the enantiomers of **2** and **41**. Previous studies have suggested that there is often a large difference in aromatase inhibition observed between the enantiomers of chiral AIs. For vorozole,[37] there is a 32-fold difference in activity, with the *S*-configuration being the most active (*S*-(+): IC_50_=1.38 nm vs *R*-(−): IC_50_=44.2 nm) and exhibiting a similar inhibition to the racemate (*R*,*S*-(±): IC_50_=2.59 nm). There is a larger 210-fold difference in aromatase inhibitory activity between the enantiomers of fadrozole hydrochloride[47] with the *S*-enantiomer proving to be the most active (*S*: IC_50_=3.2 nm vs *R*: IC_50_=680 nm). Our previous study on the enantiomers of **2** revealed a more modest fourfold difference in aromatase inhibition for the enantiomers (**2 a**: 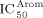
=14.3 nm vs **2 b**: 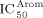
=3.2 nm), whilst for STS inhibition there was a ninefold difference between the enantiomers (**2 a**: 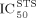
=553 nm vs **2 b**: 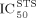
=4633 nm).

**Table 3:** In vitro inhibition of the aromatase and STS activity (nm) in JEG-3 cells by chiral sulfamates and aromatase inhibitory activity of their parent phenols

Compd	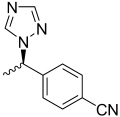		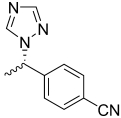	
Letrozole	AR: 0.89±0.13[a]		–	
STX64	–		STS: 1.5±0.3[b]	
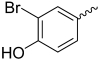	*S*-(−)-**41 a**:	AR: 3.4±0.6[c]	*R*-(+)-**41 b**:	AR: 0.6±0.1[c]
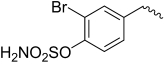	*S*-(−)-**2 a**:	AR: 14.3±2[c]	*R*-(+)-**2 b**:	AR: 3.2±0.3[c]
	*S*-(−)-**2 a**:	STS: 553±50[c]	*R*-(+)-**2 b**:	STS: 4633±551[c]
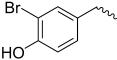	*R*-(−)-**17 a**:	AR: 4.0±0.06	*S*-(+)-**17 b**:	AR: 0.11±0.01
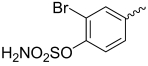	*R*-(−)-**18 a**:	AR: 30.83±2.75	*S*-(+)-**18 b**:	AR: 0.52±0.10
	*R*-(−)-**18 a**:	STS: 1150±50	*S*-(+)-**18 b**:	STS: 280±20

[a] Data taken from Wood et al.[19]

[b] Data taken from Woo et al.[21]

[c] Data taken from Wood et al.[21] Mean IC_50_ values ±SD were determined from incubations carried out in triplicate in a minimum of two separate experiments.

In the current study, there is a more significant 60-fold difference in aromatase activity between the two enantiomers of **18** (**18 a**: 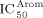
=30.83 vs **18 b**: 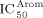
=0.52 nm) but a smaller difference (approximately fourfold) in STS inhibitory activity (**18 a**: 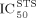
=1150 vs **18 b**: 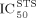
=280 nm). This increase in the difference of aromatase inhibition exhibited by each enantiomer could be a result of the increase in asymmetry of the molecule following extension of the linker length. Comparison of the aromatase inhibitory activity between the enantiomers of **2** and **18** reveals that in each case the most potent AI is the dextrorotatory enantiomer and that despite possessing different absolute configuration, the same three-dimensional relationship between the triazole ring and the *para*-cyanophenyl ring is present in the two most potent enantiomers, *R*-(+)-**2 b** and *S*-(+)-**18 b**. The spatial disposition of the heterocyle and *para*-cyano-substituted ring in **2 b** and **18 b** resembles that present in the most potent enantiomers of the chromenone-based AI series,[49] suggesting that this is the most favourable orientation of these groups for potent aromatase inhibition. For STS inhibition, there is a switch in the most potent enantiomer from the levorotatory for **2 a** to the dextrorotatory for **18 b**, and the reason for this is currently unclear.

For the parent phenols, both **17 a** and **17 b** are more potent AIs than their corresponding sulfamates, which is in accordance with the trend previously described. There is a 36-fold difference in aromatase inhibition for the two enantiomers, with the best AI being *S*-(+)-**17 b**. Significantly, the best aromatase inhibitory activity is obtained with the *S*-(+)-enantiomer of both the phenol and sulfamate providing further confirmation that this is the optimal three-dimensional relationship between the triazole ring and the *para*-cyanophenyl ring for potent inhibition.

### Molecular modelling

In order to examine the possible interaction of **18 a** and **18 b** with amino acid residues within the active site, these molecules were docked into the human aromatase crystal structure (PDB: 3EQM)[50] along with the natural substrate of the enzyme, androstenedione. For the first time, we also report the result of the docking of letrozole into the active site of aromatase. We previously verified[24] the suitability of the crystal structure for use in docking studies by removing the cocrystallised androstenedione from the substrate binding site and using the docking program GOLD[51] to dock the steroid back in. The results of this experiment indicated that the best pose of the docked androstenedione overlays the crystal structure very well.

**Figure 2:**
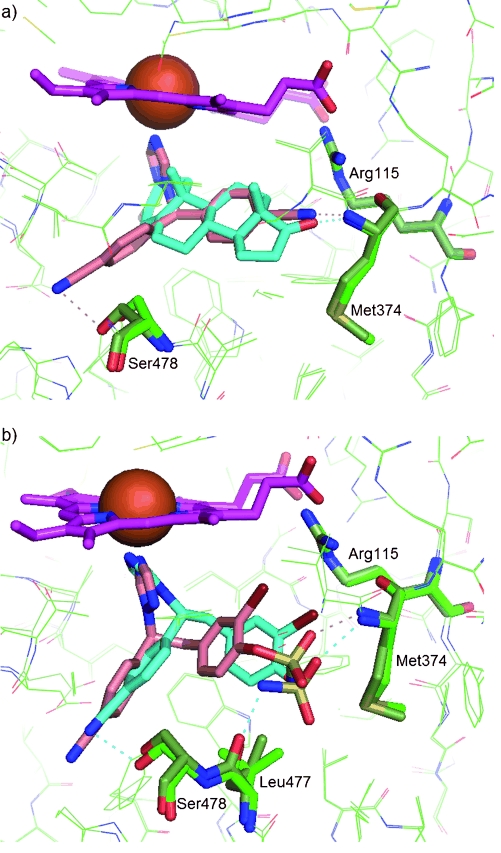
a) The docking of androstenedione and letrozole into the human aromatase crystal structure. Androstendione (cyan) is in the dark green protein and letrozole (pink) is in the light green protein. b) The docking of **18 a** and **18 b** into the aromatase active site. **18 a** (cyan) is in the light green protein and **18 b** (pink) is in the dark green protein. In both figures the haeme group is in purple with the iron represented by the orange sphere and possible hydrogen bonds are shown by dotted lines.

The results of the docking of letrozole and androstenedione into the aromatase active site are shown in Figure 2. The 17-keto oxygen atom in androstenedione is able to form a hydrogen bond with the backbone amide of Met 374 (2.75 Å), and this interaction is mimicked by one of the benzonitrile groups present in letrozole (bond distance=3.11 Å). Additionally, for the AI YM511, we recently reported[24] that the predicted docking conformation of this compound places the benzonitrile group in a similar position in the active site, and this is able to form a hydrogen-bond interaction with Met 374. Hydrogen-bond interactions with either a benzonitrile moiety or another group placed at a suitable position relative to the triazole/imidazole ring are known to be important for potent aromatase inhibitory activity in nonsteroidal AIs.[39–40] The other benzonitrile group present in letrozole is able to interact with Ser 478 (2.31 Å).

The docking of **18 a** and **18 b** into the aromatase active site is shown in Figure 2. Both **18 a** and **18 b** overlay androstenedione with their sulfamates positioned close to the 17-keto group in androstenedione. In a manner similar to letrozole, the benzonitrile groups of both **18 a** and **18 b** are able to form hydrogen-bond interactions with Ser 478 (3.54 Å and 2.27 Å, respectively), and for **18 b**, there is an additional interaction with His 480. There is no obvious structural explanation for the difference in aromatase inhibitory activity observed for the two enantiomers. To date, no human aromatase crystal structure complexed with a nonsteroidal AI has been reported. It might be more relevant and informative to dock **18 a** and **18 b** into such a crystal structure when it becomes available in the future.

**Figure 3:**
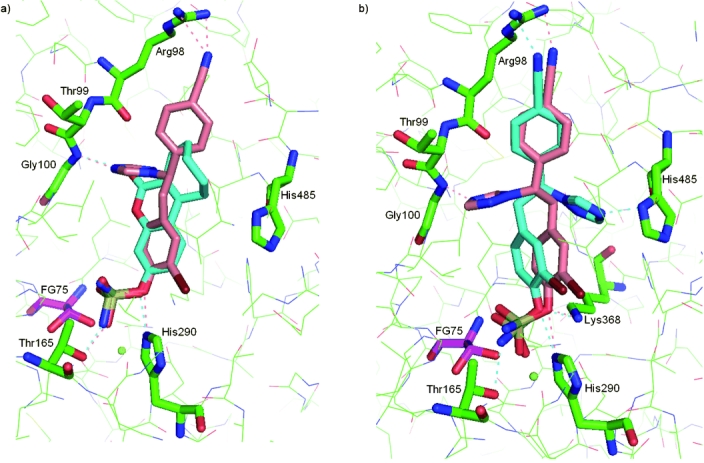
a) The docking of STX64 (cyan) and **18 a** (buff) into the crystal structure of human STS. The Ca^2+^ ion is depicted as a yellow sphere and FG75 is the *gem*-diol form of formylglycine residue 75. b) The docking of **18 b** (two poses: buff and cyan).

The docking of STX64, **18 a** and **18 b** (two poses) into the crystal structure of STS (PDB: 1P49[52]) is shown in Figure 3. Compounds **18 a** and **18 b** dock in a mode that places the sulfamate group in a satisfactory position for interaction with the *gem*-diol form of formylglycine residue 75; this is the first step in the interaction thought to be necessary for irreversible inactivation of the enzyme. In addition, the sulfamate group is predicted to be able to form favourable interactions with His 290 and Thr 165 in the active site and for **18 b** with Lys 368. Like STX64, the remainder of the skeleton of **18 a** and **18 b** occupies the hydrophobic tunnel leading to the active site. The benzonitrile groups of both **18 a** and **18 b** can form an interaction with Arg 98, and for **18 a** and one pose of **18 b**, interaction of their triazole ring with Gly 100 is possible, whilst for the other pose of **18 b**, interaction of its triazole ring with His 485 may occur.

## Conclusions

A range of DASIs structurally similar to the potent clinical AI letrozole and one compound similar to vorozole were synthesised and evaluated for aromatase and sulfatase inhibitory activity in JEG-3 cells. In order to realise molecules capable of dual inhibition, the known pharmacophore for STS inhibition (a phenol sulfamate ester) and the pharmacophore for aromatase inhibition (an N-containing heterocyclic ring) were incorporated into a single molecule.

For racemic compounds, the most potent AI identified is **29** (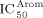
=0.12 nm), while the most potent inhibitor of STS is **40** (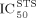
=180 nm). Consideration of the developing SAR for these derivatives reveals that extending the linker between the aromatase and STS pharmacophores is beneficial for aromatase inhibition, but this is balanced by the detrimental effects on STS inhibition resulting from extension of the linker beyond two carbon atoms. As anticipated, the addition of a halogen in the *ortho* position to the sulfamate group results in an increase in both aromatase and STS inhibitory activity. Compounds capable of potent aromatase and STS inhibition can be obtained following exchanging the *para*-cyano group for a *para*-chloro substituent, suggesting that this group is able to replicate interactions within the enzyme active site. Compound **51**, the first sulfamate-containing vorozole derivative, exhibits good inhibitory activity against aromatase meriting further investigation. Even with an IC_50_ value in the micromolar range for STS inhibition, based on established precedent, it is likely that this compound will be effective in vivo on both enzymes.

The enantiomers of **17**, the phenolic precursor of **18**, one of the most potent dual inhibitors in the current study, were separated by chiral HPLC and the absolute configuration of one enantiomer was unambiguously established using X-ray crystallography. Following conversion to the corresponding sulfamate and biological evaluation, it was established that there is a 60-fold difference in aromatase inhibition, with *S*-(+)-**18 b** being the most potent. This enantiomer has the same spatial disposition of the heterocycle and *para*-cyano-substituted ring as that found in *R*-(+)-**2 b**, the most potent enantiomer discovered in our previous study. For STS inhibition, there is a fourfold difference in inhibition with the most potent enantiomer also being *S*-(+)-**18 b**.

Molecular modelling studies indicate that both YM511 and letrozole dock into the human aromatase crystal structure with a benzonitrile group occupying a similar area of space to the 17-keto oxygen atom of androstenedione. For **18 a** and **18 b**, the sulfamate group is predicted to occupy this same area of space with their benzonitrile group able to form hydrogen bonds with Ser 478. The molecular modelling study suggests no obvious structural explanation for the difference in aromatase inhibitory activity of **18 a** and **18 b**. For STS, both **18 a** and **18 b** dock in a similar orientation to that of STX64.

These results further demonstrate the feasibility of designing a DASI based on the letrozole or vorozole templates and provide a basis for continuing pre-clinical development of such compounds for the treatment of HDBC using a multitargeted strategy.

## Experimental Section

**In vitro aromatase and sulfatase assays**: Biological assays were performed essentially as described previously.[22] The extent of in vitro inhibition of aromatase and sulfatase activities was assessed using intact monolayers of JEG-3 human choriocarcinoma cells, which were chosen because these cells constitutively express both enzymes maximally. Aromatase activity was measured using [1β-^3^H]androstenedione (30 Ci mmol^−1^, PerkinElmer Life Sciences, Waltham, MA, USA) over a 1 h period. Sulfatase activity was measured using [6,7-^3^H]E1S (50 Ci mmol^−1^, PerkinElmer Life Sciences) over a 1 h period. Each compound was tested in replicate incubations (*n*=3) using a minimum of six concentrations of the inhibitor (0.1–10 000 nm range). Mean IC_50_ values ±SD from two independent experiments are shown.

**Molecular modelling**: Models of androstenedione, letrozole, STX64, **18 a** and **18 b** were built and minimised using the Schroedinger software running under Maestro version 9.0. The GOLD docking program (version 5.0)[51] was used to dock the models into the aromatase crystal structure (PDB: 3EQM).[50] The binding site was defined as a 10 Å sphere around the androstenedione that is present in the crystal structure. A distance constraint of 2.30 Å was applied between the ligating triazole nitrogen atom of the ligand to the haeme iron atom. The ligands were then docked to the rigid enzyme a total of 25 times each and scored using the GOLDScore fitness function. To remove strain from the docked poses, the systems were put through an energy minimisation procedure using the Impact module of the Schroedinger software.

The crystal structure of human placental oestrone/DHEA sulfatase (PDB: 1P49[52]) was used for building the *gem*-diol form of steroid sulfatase (STS). This involved a point mutation of the ALS75 residue in the crystal structure to the *gem*-diol form of the structure using editing tools within the Schroedinger software. The resulting structure was then minimised with the backbone atoms fixed to allow the *gem*-diol and surrounding side chain atoms to adopt low energy confirmations. GOLD was used to dock the ligands 25 times each into the rigid protein. The docked poses were scored using the GOLDScore fitness function.

**Crystallographic data**: CCDC 806541 (**17 a**) contains the supplementary crystallographic data for this paper. These data can be obtained free of charge from The Cambridge Crystallographic Data Centre via http://www.ccdc.cam.ac.uk.

**General methods for synthesis**: All chemicals were purchased from either Aldrich Chemical Co. (Gillingham, UK) or Alfa Aesar (Heysham, UK). All organic solvents of AR grade were supplied by Fisher Scientific (Loughborough, UK). Anhydrous *N,N*-dimethylformamide (DMF), *N,N*-dimethylacetamide (DMA) and tetrahydrofuran (THF) were purchased from Aldrich. Sulfamoyl chloride was prepared by an adaptation of the method of Appel and Berger[53] and was stored under N_2_ as a solution in toluene as described by Woo et al.[54]

Thin layer chromatography (TLC) was performed on pre-coated aluminium plates (Merck, silica gel 60 F_254_). Product spots were visualised either by UV irradiation at 254 nm or by staining with either alkaline KMnO_4_ solution or 5 % *w*/*v* dodecamolybdophosphoric acid in EtOH, followed by heating. Flash column chromatography was performed using gradient elution on either pre-packed columns (Isolute) on a Flashmaster II system (Biotage) or on a Teledyne ISCO CombiFlash *R*_f_ automated flash chromatography system with RediSep *R*_f_ disposable flash columns. ^1^H and ^13^C NMR spectra were recorded on either a Jeol Delta 270 mhz or a Varian Mercury VX 400 mhz spectrometer. Chemical shifts (δ) are reported in parts per million (ppm) relative to tetramethylsilane (TMS) as an internal standard. Coupling constants (*J*) are recorded to the nearest 0.1 Hz. Mass spectra were recorded at the Mass Spectrometry Service Centre, University of Bath (UK). Fast atom bombardment (FAB) mass spectra were measured using *m*-nitrobenzyl alcohol as the matrix. Elemental analyses were performed by the Microanalysis Service, University of Bath (UK). Melting points (mp) were determined using either a Stuart Scientific SMP3 or a Stanford Research Systems Optimelt MPA100 and are uncorrected. Optical rotations were measured with a machine supplied by Optical Activity Ltd using 5 cm cells.

LC/MS was performed using a Waters 2790 machine with a ZQ MicroMass spectrometer and photodiode array (PDA) detector. The ionisation technique used was either atmospheric pressure chemical ionisation (APCI) or electrospray ionisation (ESI). A Waters “Symmetry” C18 column (packing: 3.5 μm, 4.6×100 mm) and gradient elution were used (MeCN/H_2_O, 5:95 at 0.5 mL min^−1^→95:5 at 1 mL min^−1^ over 10 min). HPLC was undertaken using a Waters 717 machine with an autosampler and PDA detector. The column used was either a Waters “Symmetry” C18 (packing: 3.5 μm, 4.6×150 mm) or a Waters “Sunfire” C18 (packing: 3.5 μm, 4.6×150 mm) with an isocratic mobile phase consisting of CH_3_CN/H_2_O (as indicated) with a flow rate of 1 mL min^−1^. Analytical chiral HPLC was performed on a Chiralpak AD-H column (250×4.6 mm, 5 μm) with MeOH as the mobile phase, a flow rate of 1.2 mL min^−1^ and a PDA detector. Semi-preparative HPLC was performed with a Waters 2525 binary gradient module and a Chiralpak AD-H (250×20 mm) semi-prep column with MeOH as the mobile phase at a flow rate of 10 mL min^−1^, injecting 1.5–2.0 mL of a 20 mg mL^−1^ solution and a run time of 25 min.

**Method A: Condensation of carbinols with triazole**: Substrate, 1,2,4-triazole and *para*-toluenesulfonic acid (*p*-TsOH), dissolved/suspended in toluene were heated at reflux with a Dean–Stark separator for 24 h. The reaction mixture was allowed to cool, and the solvent was removed in vacuo.

**Method B: Hydrogenation**: Pd/C (10 %) was added to a solution of substrate in THF/MeOH (1:1). The solution was stirred overnight under an H_2_ atmosphere (maintained using a balloon). Excess H_2_ was removed, and the reaction mixture was filtered through Celite, washing with THF and MeOH, and then the solvent was removed in vacuo.

**Method C: Sulfamoylation**: A solution of sulfamoyl chloride (H_2_NSO_2_Cl) in toluene was concentrated in vacuo at 30 °C to furnish a yellow oil, which solidified upon cooling in an ice bath. DMA and substrate were subsequently added and the mixture was allowed to warm to RT and stirred overnight. The reaction mixture was poured into H_2_O and extracted with EtOAc (2×). The organic layers were combined, washed with H_2_O (4×) and brine, dried (MgSO_4_), filtered, and the solvent was removed in vacuo.

**Method D: Removal of the TIPS protecting group**: Tetra-*n*-butylammonium fluoride (TBAF; 1 m in THF) was added to a solution of substrate in THF. After stirring for 15 min, H_2_O was added, and the solution was treated with AcOH (3 m) until colourless. The product was extracted with EtOAc, and the combined organic layers were washed with satd aq NaHCO_3_ and brine, then dried (MgSO_4_), filtered and concentrated in vacuo.

**(3-Bromo-4-(triisopropylsilyloxy)phenyl)(4-chlorophenyl)methanol (8)**: 4-ClPhMgBr (1 m in Et_2_O, 17.0 mL) was added dropwise to a solution of **7**[20] (2.00 g, 5.60 mmol) in THF (20 mL). After stirring for 2 h, the reaction was quenched by addition of H_2_O (50 mL) and 1 m aq HCl (50 mL). EtOAc (75 mL) was added, the layers were separated, and the aqueous layer was further extracted with EtOAc (75 mL). The combined organic layers were washed with H_2_O (150 mL), satd aq NaHCO_3_ (150 mL) and brine (150 mL), then dried (MgSO_4_), filtered and concentrated in vacuo. Purification using a Flashmaster II (EtOAc/hexane) gave **8** as a colourless oil (2.20 g, 84 %): ^1^H NMR (270 mhz, CDCl_3_): *δ*=1.08–1.38 (21 H, m, 6C*H*_3_, 3C*H*), 2.33 (1 H, d, *J*=3.5 Hz, O*H*), 5.69 (1 H, d, *J*=3.5 Hz, C*H*), 6.82 (1 H, d, *J*=8.4 Hz, Ar*H*), 7.07 (1 H, dd, *J*=8.4, 2.0 Hz, Ar*H*), 7.22–7.33 (4 H, m, Ar*H*), 7.48 ppm (1 H, d, *J*=2.0 Hz, Ar*H*); ^13^C NMR (100 mhz, [D_6_]DMSO): *δ*=12.9 (3CH), 17.9 (6CH_3_), 74.6 (CH), 115.1 (C), 119.4 (CH), 126.5 (CH), 127.8 (2CH), 128.6 (2CH), 131.6 (CH), 133.4 (C), 137.1 (C), 141.8 (C), 152.5 ppm (C); LC/MS (APCI−): *t*_R_=3.11 min, *m/z* (%): 469.0 (1) [*M*−H]^−^, 310.8 (100).

**2-Bromo-4-((4-chlorophenyl)(hydroxy)methyl)phenol (9)**: As method D using TBAF (9.4 mL), **8** (2.15 g, 4.59 mmol) and THF (20 mL). Purification using a Flashmaster II (EtOAc/hexane) gave **9** as a cream solid (1.32 g, 92 %): mp: 125.5–128 °C; ^1^H NMR (270 mhz, [D_6_]DMSO): *δ*=5.61 (1 H, d, *J*=4.2 Hz, C*H*), 5.94 (1 H, d, *J*=4.2 Hz, O*H*), 6.87 (1 H, d, *J*=8.2 Hz, Ar*H*), 7.11 (1 H, dd, *J*=8.2, 2.0 Hz, Ar*H*), 7.34–7.39 (4 H, m, Ar*H*), 7.42 (1 H, d, *J*=2.0 Hz, Ar*H*), 10.15 ppm (1 H, s, O*H*); ^13^C NMR (68 mhz, [D_6_]DMSO): *δ*=73.0 (CH), 109.4 (C), 116.6 (CH), 127.3 (CH), 128.5 (2CH), 128.6 (2CH), 131.1 (CH), 131.8 (C), 138.2 (C), 145.2 (C), 153.5 ppm (C); LC/MS (APCI−): *t*_R_=0.89 min, *m/z* (%): 311.0 (80) [*M*−H]^−^; HRMS-ESI: *m/z* [*M*−H]^−^ calcd for C_13_H_9_BrClO_2_: 310.9480, found: 310.9469.

**2-Bromo-4-((4-chlorophenyl)(1*H*-1,2,4-triazol-1-yl)methyl)phenol (10)**: As method A using **9** (0.80 g, 2.55 mmol), 1,2,4-triazole (1.32 g, 19.1 mmol), *p*-TsOH (0.11 g) and toluene (200 mL). The residue was dissolved in EtOAc/H_2_O (1:1, 300 mL), and the organic layer was washed with H_2_O (3×100 mL) and brine (150 mL), then dried (MgSO_4_), filtered and concetrated in vacuo. Purification using a Flashmaster II (EtOAc/hexane) gave **10** as an oil (0.87 g, 96 %); white crystals were obtained by slow evaporation of the solvent from a concentrated EtOAc solution: mp: 95–98 °C; ^1^H NMR (270 mhz, [D_6_]DMSO): *δ*=6.94 (1 H, d, *J*=8.4 Hz, Ar*H*), 7.03 (1 H, s, Ar*H*), 7.08 (1 H, dd, *J*=8.4, 2.2 Hz, Ar*H*), 7.18 (2 H, AA′BB′, Ar*H*), 7.37 (1 H, d, *J*=2.2 Hz, Ar*H*), 7.44 (2 H, AA′BB′, Ar*H*), 8.08 (1 H, s, NC*H*N), 8.60 (1 H, s, NC*H*N), 10.51 ppm (1 H, s, O*H*); ^13^C NMR (100 mhz, [D_6_]DMSO): *δ*=63.6 (CH), 109.2 (C), 116.4 (CH), 128.7 (2CH), 128.8 (CH), 129.6 (2CH), 130.6 (C), 132.6 (CH), 132.7 (C), 138.2 (C), 144.5 (CH), 152.1 (CH), 154.1 ppm (C); LC/MS (ESI−): *t*_R_=0.85 min, *m/z* (%): 362.1 (95) [*M*−H]^−^; HRMS-ESI: *m/z* [*M*−H]^−^ calcd for C_15_H_10_BrClN_3_O: 361.9690, found: 361.9700.

**2-Bromo-4-((4-chlorophenyl)(1*H*-1,2,4-triazol-1-yl)methyl)phenyl sulfamate (11)**: As method C using ClSO_2_NH_2_ (0.6 m, 3.0 mL), DMA (3 mL) and **10** (0.15 g, 0.42 mmol). Purification using a Flashmaster II (CH_2_Cl_2_/acetone) gave **11** as a white solid (0.15 g, 82 %): ^1^H NMR (270 mhz, [D_6_]DMSO): *δ*=7.20 (1 H, s, C*H*), 7.26 (2 H, AA′BB′, Ar*H*), 7.35 (1 H, dd, *J*=8.4, 2.2 Hz, Ar*H*), 7.44–7.53 (3 H, m, Ar*H*), 7.61 (1 H, d, *J*=2.2 Hz, Ar*H*), 8.12 (1 H, s, NC*H*N), 8.32 (2 H, s, N*H*_2_), 8.67 ppm (1 H, s, NC*H*N); ^13^C NMR (68 mhz, [D_6_]DMSO): *δ*=63.9 (CH), 116.6 (C), 124.2 (CH), 129.4 (C), 129.5 (2CH), 130.4 (CH), 130.5 (2CH), 133.6 (C), 137.9 (C), 139.0 (C), 145.4 (CH), 147.6 (C), 152.9 ppm (CH); LC/MS (ESI−): *t*_R_=0.89 min, *m/z* (%): 443.2 (20) [*M*−H]^−^; HRMS-ESI: *m/z* [*M*−H]^−^ calcd for C_15_H_11_BrClN_4_O_3_S: 440.9429, found: 440.9421.

**4-(2-(4-Hydroxyphenyl)-1-(1*H*-1,2,4-triazol-1-yl)ethyl)benzonitrile (13)**: *n*BuLi (1.95 m, 3.00 mL) was added dropwise to a cooled (−78 °C) solution of 4-((1*H*-1,2,4-triazol-1-yl)methyl)benzonitrile[19] (0.95 g, 5.16 mmol) in THF (50 mL). After 30 min, a solution of **12**[30] (2.30 g, 6.71 mmol) in THF (25 mL) was added, and the mixture was stirred for 30 min. The reaction mixture was allowed to warm to RT and stirred for 4 h. The reaction was quenched by cautious addition of satd aq NH_4_Cl. The layers were separated and the organic was dried (MgSO_4_), filtered and concentrated in vacuo. The crude intermediate was purified using a CombiFlash *R*_f_ (EtOAc/PE) and used without further purification. Deprotection was achieved using method D with TBAF (2.5 mL) and a solution of the intermediate in THF (10 mL). Purification using a Combiflash *R*_f_ (EtOAc/PE) gave **13** as a white solid (0.35 g, 23 %): mp: 221.5–223.5 °C; ^1^H NMR (270 mhz, [D_6_]DMSO): *δ*=3.32 (1 H, dd, *J*=14.0, 5.8 Hz, CH*H*), 3.51 (1 H, dd, *J*=14.0, 9.6 Hz, CH*H*), 5.93 (1 H, dd, *J*=9.6, 5.8 Hz, C*H*), 6.58 (2 H, AA′BB′, Ar*H*), 6.92 (2 H, AA′BB′, Ar*H*), 7.61 (2 H, AA′BB′, Ar*H*), 7.83 (2 H, AA′BB′, Ar*H*), 8.00 (1 H, s, NC*H*N), 8.56 (1 H, s, NC*H*N), 9.24 ppm (1 H, s, O*H*); ^13^C NMR (100 mhz, [D_6_]DMSO): *δ*=39.3 (CH_2_), 63.7 (CH), 110.8 (C), 115.1 (2CH), 118.6 (C), 126.9 (C), 128.2 (2CH), 129.9 (2CH), 132.5 (2CH), 144.1 (CH), 145.0 (C), 151.7 (CH), 156.0 ppm (C); LC/MS (ESI+): *t*_R_=1.31 min, *m/z* (%): 291.1 (100) [*M*+H]^+^; HRMS-ESI: *m/z* [*M*+H]^+^ calcd for C_17_H_15_N_4_O: 291.1240, found: 291.1234; Anal. calcd for C_17_H_14_N_4_O: C 70.33, H 4.86, N 19.30, found: C 70.30, H 4.88, N 19.20.

**4-(2-(4-Cyanophenyl)-2-(1*H*-1,2,4-triazol-1-yl)ethyl)phenyl sulfamate (14)**: As method C using ClSO_2_NH_2_ (0.57 m, 6.8 mL), DMA (1 mL) and a solution of **13** (0.19 g, 0.66 mmol) in DMA (1 mL). Purification using a Combiflash *R*_f_ (CHCl_3_/acetone) gave **14** as a white foam (0.19 g, 88 %): ^1^H NMR (270 mhz, [D_6_]DMSO): *δ*=3.49 (1 H, dd, *J*=14.0, 5.5 Hz, CH*H*), 3.68 (1 H, dd, *J*=14.0, 9.9 Hz, CH*H*), 6.08 (1 H, dd, *J*=14.0, 9.9 Hz, C*H*), 7.12 (2 H, AA′BB′, Ar*H*), 7.25 (2 H, AA′BB′, Ar*H*), 7.64 (2 H, AA′BB′, Ar*H*), 7.86 (2 H, AA′BB′, Ar*H*), 7.96 (2 H, s, N*H*_2_), 8.02 (1 H, s, NC*H*N), 8.62 ppm (1 H, s, NC*H*N); ^13^C NMR (100 mhz, [D_6_]DMSO): *δ*=39.1 (CH_2_), 63.1 (CH), 111.0 (C), 118.5 (C), 121.8 (2CH), 128.2 (2CH), 130.2 (2CH), 132.6 (2CH), 135.3 (C), 144.1 (C), 144.8 (CH), 148.9 (C), 151.9 ppm (CH); LC/MS (ESI+): *t*_R_=1.27 min, *m/z* (%): 370.1 (100) [*M*+H]^+^; HRMS-ESI: *m/z* [*M*+H]^+^ calcd for C_17_H_16_N_5_O_3_S: 370.0968, found: 370.0958.

**(3-Bromo-4-(triisopropylsilyloxy)phenyl)methanol (15)**: A solution of NaBH_4_ (0.19 g, 5.00 mmol) in H_2_O (2 mL) was added dropwise to a solution of **7** (0.70 g, 1.96 mmol) in EtOH (6 mL). After stirring for 15 min, the reaction was quenched by cautious addition of aq HCl (3 m, 15 mL), EtOAc (15 mL) and H_2_O (15 mL). The layers were separated, and the aqueous was extracted with EtOAc (30 mL). The combined organic layers were washed with H_2_O (2×50 mL) and satd aq NaHCO_3_ (50 mL), then dried (MgSO_4_), filtered and concentrated in vacuo. Purification using a Flashmaster II (EtOAc/hexane) gave **15** as a colourless oil (0.57 g, 81 %): ^1^H NMR (270 mhz, CDCl_3_): *δ*=1.10–1.37 (21 H, m, 6C*H*_3_, 3C*H*), 1.58 (1 H, t, *J*=5.7 Hz, O*H*), 4.57 (1 H, d, *J*=5.7 Hz, C*H*_2_), 6.85 (1 H, d, *J*=8.2 Hz, Ar*H*), 7.13 (1 H, dd, *J*=8.2, 2.2 Hz, Ar*H*), 7.52 ppm (1 H, d, *J*=2.2 Hz, Ar*H*); ^13^C NMR (100 mhz, CDCl_3_): *δ*=12.9 (3CH), 18.0 (6CH_3_), 64.4 (CH_2_), 115.0 (C), 119.5 (CH), 127.1 (CH), 132.2 (CH), 134.6 (C), 152.5 ppm (C); LC/MS (ESI+): *t*_R_=2.02 min, *m/z* (%): 341.2 (100) [*M*−H_2_O]^+^.

**(2-Bromo-4-(chloromethyl)phenoxy)triisopropylsilane (16)**: SOCl_2_ (0.98 g, 7.10 mmol) was added dropwise to a solution of **15** (0.51 g, 1.42 mmol) in CH_2_Cl_2_ (8 mL). After 1 h, the solvent was removed in vacuo, and the residue was redissolved in CH_2_Cl_2_ (15 mL) and evaporated; this process was repeated twice more to give **16** as a white solid (0.54 g, 100 %): ^1^H NMR (270 mhz, CDCl_3_): *δ*=1.10–1.38 (21 H, m, 6C*H*_3_, 3C*H*), 4.49 (2 H, s, C*H*_2_), 6.83 (1 H, d, *J*=8.4 Hz, Ar*H*), 7.15 (1 H, dd, *J*=8.4, 2.5 Hz, Ar*H*), 7.53 ppm (1 H, d, *J*=2.5 Hz, Ar*H*); ^13^C NMR (68 mhz, CDCl_3_): *δ*=13.0 (3CH), 18.0 (6CH_3_), 45.4 (CH_2_), 115.1 (C), 119.6 (CH), 128.7 (CH), 131.2 (C), 133.8 (CH), 153.2 ppm (C); LC/MS (ESI+): *t*_R_=3.30 min, *m/z* (%): 341.2 (40) [*M*−Cl]^+^, 236 (90), 185 (100).

**4-(2-(3-Bromo-4-hydroxyphenyl)-1-(1*H*-1,2,4-triazol-1-yl)ethyl)benzonitrile (17)**: *n*BuLi (2.12 m, 3.80 mL) was added dropwise to a cooled (−78 °C) solution of 4-((1*H*-1,2,4-triazol-1-yl)methyl)benzonitrile[19] (1.68 g, 9.13 mmol) in THF (50 mL). After 30 min, a solution of **16** (3.79 g, 10.0 mmol) in THF (10 mL) was added, and the mixture was stirred for 1 h. The reaction mixture was allowed to warm to RT and stirred for 2 h. The reaction was quenched by cautious addition of satd aq NH_4_Cl. The layers were separated, and the organic layer was dried (MgSO_4_), filtered and concentrated in vacuo. The crude intermediate was purified using a Flashmaster II (EtOAc/hexane) and used without further purification. Deprotection was achieved using method D with TBAF (7.6 mL) and a solution of the intermediate in THF (30 mL). Purification using a Flashmaster II (EtOAc/PE) gave **17** as a white solid (2.04 g, 81 %): mp: 186–188.5 °C; ^1^H NMR (270 mhz, [D_6_]DMSO): *δ*=3.30–3.39 (1 H, m, C*H*H), 3.51 (1 H, dd, *J*=13.9, 9.9 Hz, CH*H*), 5.98 (1 H, dd, *J*=9.9, 5.7 Hz, C*H*), 6.74 (1 H, d, *J*=8.2 Hz, Ar*H*), 6.88 (1 H, dd, *J*=8.2, 2.0 Hz, Ar*H*), 7.35 (1 H, *J*=2.0 Hz, Ar*H*), 7.62 (2 H, AA′BB′, Ar*H*), 7.84 (2 H, AA′BB′, Ar*H*), 8.02 (1 H, s, NC*H*N), 8.59 (1 H, s, NC*H*N), 10.07 ppm (1 H, br s, O*H*); ^13^C NMR (100 mhz, [D_6_]DMSO): *δ*=39.1 (CH_2_), 63.8 (CH), 109.5 (C), 111.3 (C), 116.5 (CH), 119.0 (C), 128.7 (2CH), 129.4 (C), 129.7 (CH), 133.0 (2CH), 133.6 (CH), 144.6 (CH), 145.3 (C), 152.3 (CH), 153.2 ppm (C); LC/MS (ESI+): *t*_R_=1.39 min, *m/z* (%): 369.0 (100) [*M*+H]^+^; HRMS-ESI: *m/z* [*M*+H]^+^ calcd for C_17_H_14_BrN_4_O: 369.0346, found: 369.0334.

**2-Bromo-4-(2-(4-cyanophenyl)-2-(1*H*-1,2,4-triazol-1-yl)ethyl)phenyl sulfamate (18)**: As method C using ClSO_2_NH_2_ (0.6 m, 4.0 mL), DMA (2 mL) and **17** (0.15 g, 0.41 mmol). Purification using a Flashmaster II (CH_2_Cl_2_/acetone) gave **18** as a white solid (0.16 g, 88 %): ^1^H NMR (270 mhz, [D_6_]DMSO): *δ*=3.49 (1 H, dd, *J*=14.0, 5.5 Hz, CH*H*), 3.67 (1 H, dd, *J*=14.0, 10.2 Hz, CH*H*), 6.12 (1 H, dd, *J*=10.2, 5.5 Hz, C*H*), 7.21 (1 H, dd, *J*=8.6, 1.9 Hz, Ar*H*), 7.32 (1 H, d, *J*=8.6 Hz, Ar*H*), 7.62–7.68 (3 H, m, Ar*H*), 7.86 (2 H, AA′BB′, Ar*H*), 8.04 (1 H, s, NC*H*N), 8.23 (2 H, s, N*H*_2_), 8.64 ppm (1 H, s, NC*H*N); ^13^C NMR (100 mhz, [D_6_]DMSO): *δ*=38.6 (CH_2_), 62.8 (CH), 111.0 (C), 115.5 (C), 118.4 (C), 122.9 (CH), 128.2 (2CH), 129.5 (C), 132.7 (2CH), 133.9 (CH), 137.1 (C), 144.3 (CH), 144.7 (C), 146.1 (C), 152.0 ppm (CH); LC/MS (ESI−): *t*_R_=0.78 min, *m/z* (%): 446.1 (100) [*M*−H]^−^; HRMS-ESI: *m/z* [*M*+H]^+^ calcd for C_17_H_15_BrN_5_O_3_S: 448.0073, found: 448.0074.

**4-(1-(1*H*-1,2,4-Triazol-1-yl)-3-(4-(triisopropylsilyloxy)phenyl)propyl)benzonitrile (20)**: *n*BuLi (1.75 m, 0.55 mL) was added dropwise to a cooled (−78 °C) solution of 4-((1*H*-1,2,4-triazol-1-yl)methyl)benzonitrile[19] (0.16 g, 0.87 mmol) in THF (4 mL). After 30 min, a solution of **19**[33] (0.40 g, 1.12 mmol) in THF (1 mL) was added, and the mixture was stirred for 2 h. The reaction mixture was allowed to warm to RT and stirred for 1 h. The reaction was quenched by cautious addition of satd aq NH_4_Cl. The layers were separated, and the organic layer was dried (MgSO_4_), filtered and concentrated in vacuo. Purification using a Flashmaster II (EtOAc/hexane) gave **20** as a colourless oil (0.22 g, 55 %): ^1^H NMR (270 mhz, CDCl_3_): *δ*=1.04–1.32 (21 H, m, 6C*H*_3_, 3C*H*), 2.30–2.84 (4 H, m, C*H*_2_C*H*_2_), 5.23 (1 H, dd, *J*=9.4, 4.4 Hz, C*H*), 6.80 (2 H, AA′BB′, Ar*H*), 6.91 (2 H, AA′BB′, Ar*H*), 7.37 (2 H, AA′BB′, Ar*H*), 7.62 (2 H, AA′BB′, Ar*H*), 8.02 (1 H, s, NC*H*N), 8.05 ppm (1 H, s, NC*H*N); ^13^C NMR (100 mhz, CDCl_3_): *δ*=12.6 (3CH), 17.9 (6CH_3_), 31.2 (CH_2_), 36.5 (CH_2_), 62.4 (CH), 112.4 (C), 118.2 (C), 120.2 (2CH), 127.6 (2CH), 129.2 (2CH), 131.7 (C), 132.7 (2CH), 143.1 (CH), 144.3(C), 152.5 (CH), 154.7 ppm (C); LC/MS (ESI+): *t*_R_=5.11 min, *m/z* (%): 461.2 (80) [*M*+H]^+^, 263.0 (100); HRMS-ESI: *m/z* [*M*+H]^+^ calcd for C_27_H_37_N_4_OSi: 461.2731, found: 461.2711.

**4-(3-(4-Hydroxyphenyl)-1-(1*H*-1,2,4-triazol-1-yl)propyl)benzonitrile (21)**: As method D using TBAF (0.42 mL), **20** (0.18 g, 0.39 mmol) and THF (3 mL). Purification using a Flashmaster II (EtOAc/hexane) gave **21** as a white foam (0.11 g, 95 %): ^1^H NMR (270 mhz, CDCl_3_): *δ*=2.26–2.82 (4 H, m, C*H*_2_C*H*_2_), 5.27 (1 H, dd, *J*=9.6, 5.2 Hz, C*H*), 6.75 (2 H, AA′BB′, Ar*H*), 6.87 (2 H, AA′BB′, Ar*H*), 7.38 (2 H, AA′BB′, Ar*H*), 7.59 (2 H, AA′BB′, Ar*H*), 8.04 (1 H, s, NC*H*N), 8.13 ppm (1 H, s, NC*H*N); ^13^C NMR (100 mhz, [D_6_]DMSO): *δ*=30.8 (CH_2_), 35.9 (CH_2_), 61.7 (CH), 110.8 (C), 115.2 (2CH), 118.5 (C), 127.9 (2CH), 129.1 (2CH), 130.3 (C), 132.7 (2CH), 144.1 (CH), 145.3 (C), 151.9 (CH), 155.6 ppm (C); LC/MS (ESI+): *t*_R_=3.54 min, *m/z* (%): 305.2 (100) [*M*+H]^+^; HRMS-ESI: *m/z* [*M*+H]^+^ calcd for C_18_H_17_N_4_O: 305.1397, found: 305.1389.

**4-(3-(4-Cyanophenyl)-3-(1*H*-1,2,4-triazol-1-yl)propyl)phenyl sulfamate (22)**: As method C using ClSO_2_NH_2_ (0.5 m, 5.0 mL), DMA (3 mL) and **21** (0.14 g, 0.46 mmol). Purification using a CombiFlash *R*_f_ (CH_2_Cl_2_/acetone) gave **22** as a white foam (0.16 g, 91 %): ^1^H NMR (270 mhz, [D_6_]DMSO): *δ*=2.42–2.78 (4 H, m, C*H*_2_C*H*_2_), 5.71–5.75 (1 H, m, C*H*), 7.18 (2 H, AA′BB′, Ar*H*), 7.25 (2 H, AA′BB′, Ar*H*), 7.57 (2 H, AA′BB′, Ar*H*), 7.85 (2 H, AA′BB′, Ar*H*), 7.94 (2 H, br s, N*H*_2_), 8.08 (1 H, s, NC*H*N), 8.79 ppm (1 H, s, NC*H*N); ^13^C NMR (100 mhz, [D_6_]DMSO): *δ*=31.1 (CH_2_), 35.4 (CH_2_), 61.8 (CH), 110.9 (C), 118.5 (C), 122.1 (2CH), 127.9 (2CH), 129.5 (2CH), 132.7 (2CH), 138.8 (C), 144.2 (CH), 145.2 (C), 148.5 (C), 152.0 ppm (CH); LC/MS (ESI+): *t*_R_=1.26 min, *m/z* (%): 384.3 (100) [*M*+H]^+^; HRMS-ESI: *m/z* [*M*+H]^+^ calcd for C_18_H_18_N_5_O_3_S: 384.1108, found: 384.1125.

**Methyl 2-(3-bromo-4-(triisopropylsilyloxy)phenyl)acetate (24)**: Imidazole (8.77 g, 0.13 mol) and triisopropylsilyl chloride (TIPSCl; 14.32 g, 74.2 mmol) were sequentially added to a pale yellow solution of **23**[23] (15.80 g, 64.5 mmol) in DMF (50 mL). The reaction mixture was stirred overnight, then poured into H_2_O (100 mL) and extracted with EtOAc (3×50 mL). The combined organic extracts were washed with H_2_O (3×60 mL) and brine (60 mL), then dried (MgSO_4_), filtered and concentrated in vacuo. Purification using a CombiFlash *R*_f_ (EtOAc/PE) gave **24** as a colourless oil (22.77 g, 88 %): ^1^H NMR (270 mhz, CDCl_3_): *δ*=1.07–1.37 (21 H, m, 6C*H*_3_, 3C*H*), 3.51 (2 H, s, C*H*_2_), 3.68 (3 H, s, C*H*_3_), 6.81 (1 H, d, *J*=8.3 Hz, Ar*H*), 7.04 (1 H, dd, *J*=8.3, 2.2 Hz, Ar*H*), 7.42 ppm (1 H, d, *J*=2.2 Hz, Ar*H*); ^13^C NMR (100 mhz, CDCl_3_): *δ*=12.9 (3CH), 18.0 (6CH_3_), 39.8 (CH_2_), 52.1 (CH_3_), 114.9 (C), 119.4 (CH), 129.5 (C), 129.0 (CH), 134.0 (CH), 152.1 (C), 171.8 ppm (C).

**2-(3-Bromo-4-(triisopropylsilyloxy)phenyl)ethanol (25)**: LiBH_4_ (3.04 g, 0.14 mol) and B(OMe)_3_ (0.57 g, 5.84 mmol) were sequentially added to a stirred solution of **24** (22.10 g, 55.1 mmol) in Et_2_O (150 mL). The reaction mixture was stirred for 48 h and then diluted with Et_2_O (150 mL) and quenched by cautious addition of H_2_O (75 mL) and HCl (3 m, 75 mL). The layers were separated, and the organic layer was washed with brine (150 mL), then dried (MgSO_4_), filtered and concentrated in vacuo. Purification using a CombiFlash *R*_f_ (EtOAc/PE) gave **25** as a colourless oil (18.72 g, 91 %): ^1^H NMR (270 mhz, CDCl_3_): *δ*=1.09–1.39 (22 H, m, 3C*H*, 6C*H*_3,_ O*H*), 2.76 (2 H, t, *J*=6.3 Hz, C*H*_2_), 3.80 (2 H, q, *J*=6.3 Hz, C*H*_2_OH), 6.80 (1 H, d, *J*=8.3 Hz, Ar*H*), 6.98 (1 H, dd, *J*=8.3, 2.2 Hz, Ar*H*), 7.37 ppm (1 H, d, *J*=2.2 Hz, Ar*H*); ^13^C NMR (100 mhz, CDCl_3_): *δ*=12.9 (3CH), 18.0 (6CH_3_), 37.9 (CH_2_), 63.5 (CH_2_), 115.0 (C), 119.5 (CH), 128.7 (CH), 132.1 (C), 133.7 (CH), 151.5 ppm (C); LC/MS (ESI+): *t*_R_=6.14 min, *m/z* (%): 373.2 (100) [*M*+H]^+^; HRMS-ESI: *m/z* [*M*+H]^+^ calcd for C_17_H_30_BrO_2_Si: 373.1193, found: 373.1181.

**(2-Bromo-4-(2-bromoethyl)phenoxy)triisopropylsilane (26)**: PPh_3_ (5.44 g, 20.7 mmol), imidazole (1.41 g, 20.7 mmol) and Br_2_ (3.22 g, 20.1 mmol) were sequentially added to a solution of **25** (2.50 g, 6.70 mmol) in Et_2_O/CH_3_CN (55 mL:19 mL). After stirring for 3 h, the reaction mixture was filtered, washing with Et_2_O. The organic layers were combined and washed with brine, then dried (MgSO_4_), filtered and concentrated in vacuo. Purification using a CombiFlash *R*_f_ (EtOAc/PE) gave **26** as a colourless oil (2.58 g, 88 %): ^1^H NMR (270 mhz, CDCl_3_): *δ*=1.08–1.37 (21 H, m, 3C*H*, 6C*H*_3_), 3.04 (2 H, t, *J*=7.7 Hz, C*H*_2_), 3.50 (2 H, t, *J*=7.7 Hz, C*H*_2_), 6.81 (1 H, d, *J*=8.3 Hz, Ar*H*), 6.96 (1 H, dd, *J*=8.3, 2.2 Hz, Ar*H*), 7.35 ppm (1 H, d, *J*=2.2 Hz, Ar*H*).

**4-(3-(3-Bromo-4-(triisopropylsilyloxy)phenyl)-1-(1*H*-1,2,4-triazol-1-yl)propyl)benzonitrile (27)**: *n*BuLi (1.75 m, 2.63 mL) was added dropwise to a cooled (−78 °C) solution of 4-((1*H*-1,2,4-triazol-1-yl)methyl)benzonitrile[19] (0.77 g, 4.18 mmol) in THF (25 mL). After 30 min, a solution of **26** (2.20 g, 5.05 mmol) in THF (5 mL) was added, and the mixture was stirred for 2 h. The reaction mixture was allowed to warm to RT and stirred for 1 h. The reaction was quenched by cautious addition of satd aq NH_4_Cl. The layers were separated, and the organic was dried (MgSO_4_), filtered and concentrated in vacuo. Purification using a CombiFlash *R*_f_ (EtOAc/PE) gave **27** as a yellow oil (0.91 g, 40 %): ^1^H NMR (270 mhz, CDCl_3_): *δ*=1.08–1.38 (21 H, m, 6C*H*_3_, 3C*H*), 2.30–2.53 (3 H, m, 3C*H*H), 2.72–2.82 (1 H, m, C*H*H), 5.26 (1 H, dd, *J*=9.6, 5.2 Hz, C*H*), 6.79–6.86 (2 H, m, Ar*H*), 7.24–7.26 (1 H, m, Ar*H*), 7.39 (2 H, AA′BB′, Ar*H*), 7.62 (2 H, AA′BB′, Ar*H*), 8.02 (1 H, s, NC*H*N), 8.08 ppm (1 H, s, NC*H*N); ^13^C NMR (100 mhz, CDCl_3_): *δ*=12.9 (3CH), 17.9 (6CH_3_), 30.9 (CH_2_), 36.4 (CH_2_), 62.5 (CH), 112.5 (C), 115.2 (C), 118.2 (C), 119.7 (CH), 127.6 (2CH), 128.1 (CH), 132.7 (2CH), 133.0 (CH), 133.1 (C), 143.0 (CH), 144.1 (C), 151.6 (C), 152.5 ppm (CH); LC/MS (ESI+): *t*_R_=6.62 min, *m/z* (%): 539.5 (100) [*M*+H]^+^; HRMS-ESI: *m/z* [*M*+H]^+^ calcd for C_27_H_36_BrN_4_OSi: 539.1836, found: 539.1816.

**4-(3-(3-Bromo-4-hydroxyphenyl)-1-(1*H*-1,2,4-triazol-1-yl)propyl)benzonitrile (28)**: As method D using TBAF (1.72 mL), **27** (0.83 g, 1.54 mmol) and THF (10 mL). Purification using a CombiFlash *R*_f_ (CH_2_Cl_2_/acetone) gave **28** as a foam (0.46 g, 79 %): ^1^H NMR (270 mhz, CDCl_3_): *δ*=2.20–2.80 (4 H, m, C*H*_2_C*H*_2_), 5.66 (1 H, dd, *J*=9.6, 5.8 Hz, C*H*), 6.84 (1 H, d, *J*=8.3 Hz, Ar*H*), 6.94 (1 H, dd, *J*=8.3, 1.9 Hz, Ar*H*), 7.26 (1 H, d, *J*=1.9 Hz, Ar*H*), 7.54 (2 H, AA′BB′, Ar*H*), 7.83 (2 H, AA′BB′, Ar*H*), 8.06 (1 H, s, NC*H*N), 8.77 (1 H, s, NC*H*N), 10.06 ppm (1 H, br s, O*H*); ^13^C NMR (100 mhz, [D_6_]DMSO): *δ*=35.5 (CH_2_), 38.9 (CH_2_), 61.7 (CH), 109.1 (C), 110.8 (C), 116.3 (CH), 118.5 (C), 127.9 (2CH), 128.5 (CH), 132.3 (CH), 132.4 (C), 132.7 (2CH), 144.1 (CH), 145.2 (C), 151.9 (CH), 152.3 ppm (C); LC/MS (ESI+): *t*_R_=1.35 min, *m/z* (%): 383.1 (100) [*M*+H]^+^; HRMS-ESI: *m/z* [*M*+H]^+^ calcd for C_18_H_16_BrN_4_O: 383.0502, found: 383.0497.

**2-Bromo-4-(3-(4-cyanophenyl)-3-(1*H*-1,2,4-triazol-1-yl)propyl)phenyl sulfamate (29)**: As method C using ClSO_2_NH_2_ (0.57 m, 4.0 mL), DMA (2.5 mL) and **28** (0.16 g, 0.42 mmol). Purification using a CombiFlash *R*_f_ (CH_2_Cl_2_/acetone) gave **29** as a white solid (0.13 g, 67 %): ^1^H NMR (270 mhz, [D_6_]DMSO): *δ*=2.36–2.72 (4 H, m, 2C*H*_2_), 5.74 (1 H, dd, *J*=9.2, 5.2 Hz, C*H*), 7.24 (1 H, dd, *J*=8.3, 2.2 Hz, Ar*H*), 7.38 (1 H, d, *J*=8.3 Hz, Ar*H*), 7.53–7.59 (3 H, m, Ar*H*), 7.85 (2 H, AA′BB′, Ar*H*), 8.06 (1 H, s, NC*H*N), 8.21 (2 H, br s, N*H*_2_), 8.79 ppm (1 H, s, NC*H*N); ^13^C NMR (100 mhz, [D_6_]DMSO): *δ*=30.8 (CH_2_), 35.1 (CH_2_), 61.9 (CH), 110.9 (C), 115.7 (C), 118.5 (C), 123.1 (CH), 128.0 (2CH), 128.8 (CH), 132.7 (2CH), 133.1 (CH), 140.7 (C), 144.1 (C), 145.1 (CH), 145.7 (C), 151.9 ppm (CH); LC/MS (ESI+): *t*_R_=1.30 min, *m/z* (%): 462.0 (100) [*M*+H]^+^; HRMS-ESI: *m/z* [*M*+H]^+^ calcd for C_18_H_17_BrN_5_O_3_S: 462.0230, found: 462.0216.

**2-(4-(Triisopropylsilyloxy)phenyl)acetaldehyde (31)**: A solution of **30**[33] (0.25 g, 0.85 mmol) in CH_2_Cl_2_ (1 mL) was added to a suspension of Dess–Martin reagent (0.40 g, 0.94 mmol) in CH_2_Cl_2_ (5 mL). After stirring for 1 h, Et_2_O (5 mL), satd aq NaHCO_3_ (5 mL) and satd aq Na_2_S_2_O_3_ (5 mL) were added and stirring continued for 10 min. The layers were separated, and the aqueous layer was extracted with Et_2_O (10 mL). The combined organic layers were washed with satd aq NaHCO_3_ (20 mL), H_2_O (20 mL) and brine (20 mL), then dried (MgSO_4_), filtered and concentrated in vacuo. Purification using a CombiFlash *R*_f_ (EtOAc/PE) gave **31** as a yellow oil (0.20 g, 81 %): ^1^H NMR (270 mhz, CDCl_3_): *δ*=1.05–1.31 (21 H, m, 3C*H*, 6C*H*_3_), 3.59 (2 H, d, *J*=2.2 Hz, C*H*_2_), 6.86 (2 H, AA′BB′, Ar*H*), 7.05 (2 H, AA′BB′, Ar*H*), 9.70 ppm (1 H, t, *J*=2.5 Hz, C*H*O); ^13^C NMR (100 mhz, CDCl_3_): *δ*=12.7 (3CH), 18.0 (6CH_3_), 49.9 (CH_2_), 120.5 (2CH), 124.1 (C), 130.7 (2CH), 155.6 (C), 199.9 ppm (CH).

**1-(4-Chlorophenyl)-2-(4-(triisopropylsilyloxy)phenyl)ethanol (32)**: 4-ClPhMgBr (1 m in Et_2_O, 8.25 mL) was added to a solution of **31** (1.20 g, 4.10 mmol) in Et_2_O (20 mL). After stirring for 2 h, the reaction was quenched by addition of H_2_O (10 mL) and 2 m HCl (10 mL). The product was extracted with Et_2_O (2×30 mL), and the combined organic layers were washed with brine (40 mL), then dried (MgSO_4_), filtered and concentrated in vacuo. Purification using a CombiFlash *R*_f_ (EtOAc/PE) gave **32** as a white solid (2.09 g, 90 %): mp: 71–73 °C; ^1^H NMR (270 mhz, CDCl_3_): *δ*=1.06–1.32 (21 H, m, 3C*H*, 6C*H*_3_), 1.99 (1 H, d, *J*=3.0 Hz, O*H*), 2.86–2.93 (2 H, m, C*H*_2_), 4.76–4.86 (1 H, m, C*H*), 6.79 (2 H, AA′BB′, Ar*H*), 6.95 (2 H, AA′BB′, Ar*H*), 7.14–7.32 ppm (4 H, m, Ar*H*); ^13^C NMR (100 mhz, CDCl_3_): *δ*=12.6 (3CH), 17.9 (6CH_3_), 45.3 (CH_2_), 74.7 (CH), 120.0 (2CH), 127.3 (2CH), 128.4 (2CH), 129.6 (C), 130.4 (2CH), 133.1 (C), 142.1 (C), 154.9 ppm (C); HRMS-ESI: *m/z* [*M*+Na]^+^ calcd for C_23_H_32_BrClNaO_2_Si: 505.0936, found: 505.0920.

**(4-(2-Chloro-2-(4-chlorophenyl)ethyl)phenoxy)triisopropylsilane (33)**: SOCl_2_ (1.90 g, 1.01 mmol) and DMF (5 drops) were added to a solution of **32** (1.62 g, 4.00 mmol) in CH_2_Cl_2_ (40 mL). After 1 h, the solvent was removed in vacuo, the residue was redissolved in CH_2_Cl_2_, and the process was repeated. Purification using a CombiFlash *R*_f_ (EtOAc/PE) gave **33** as a yellow oil (1.55 g, 92 %): ^1^H NMR (270 mhz, CDCl_3_): *δ*=1.07–1.31 (21 H, m, 3C*H*, 6C*H*_3_), 3.18 (1 H, dd, *J*=13.8, 7.7 Hz, C*H*H), 3.34 (1 H, dd, *J*=13.8, 6.9 Hz, C*H*H), 4.94 (1 H, t, *J*=6.6 Hz, C*H*), 6.75 (2 H, AA′BB′, Ar*H*), 6.86 (2 H, AA′BB′, Ar*H*), 7.15–7.29 ppm (4 H, m, Ar*H*); ^13^C NMR (68 mhz, CDCl_3_): *δ*=12.7 (3CH), 18.0 (6CH_3_), 46.1 (CH_2_), 63.4 (CH), 120.0 (2CH), 128.6 (2CH), 128.7 (2CH), 129.5 (C), 130.5 (2CH), 134.0 (C), 139.6 (C), 155.1 ppm (C).

**4-(2-(4-Chlorophenyl)-2-(1*H*-1,2,4-triazol-1-yl)ethyl)phenol (34)**: 1,2,4-Triazole (0.63 g, 9.13 mmol), K_2_CO_3_ (0.60 g, 4.35 mmol) and KI (0.060 g, 0.36 mmol) were sequentially added to a solution of **33** (1.54 g, 3.64 mmol) in acetone (70 mL). The reaction mixture was heated at 55 °C and monitored by TLC with additional portions of triazole added when required. After 4 d, the reaction mixture was allowed to cool, and the solvent was removed in vacuo. The residue was redissolved in H_2_O/EtOAc (1:1, 160 mL), the layers were separated, and the aqueous layer extracted with EtOAc (80 mL). The organic layers were combined, washed with brine (100 mL), then dried (MgSO_4_), filtered and concentrated in vacuo. Purification using a CombiFlash *R*_f_ (PE/EtOAc) gave **34** as a white crystalline solid (0.53 g, 49 %): mp: 99–101 °C; ^1^H NMR (270 mhz, [D_6_]DMSO): *δ*=3.29 (1 H, dd, *J*=14.0, 5.8 Hz, C*H*H), 3.49 (1 H, dd, *J*=14.0, 9.6 Hz, C*H*H), 5.83 (1 H, dd, *J*=9.6, 5.8 Hz, C*H*), 6.58 (2 H, AA′BB′, Ar*H*), 6.91 (2 H, AA′BB′, Ar*H*), 7.39–7.50 (4 H, m, Ar*H*), 7.98 (1 H, s, NC*H*N), 8.55 (1 H, s, NC*H*N), 9.24 ppm (1 H, s, O*H*); ^13^C NMR (100 mhz, [D_6_]DMSO): *δ*=39.5 (CH_2_), 63.5 (CH), 115.0 (2CH), 127.2 (C), 128.5 (2CH), 129.1 (2CH), 129.9 (2CH), 132.6 (C), 138.8 (C), 143.8 (CH), 151.5 (CH), 155.9 ppm (C); LC/MS (ESI+): *t*_R_=1.54 min, *m/z* (%): 300.2 (100) [*M*+H]^+^, 231.1 (70); HRMS-ESI: *m/z* [*M*+H]^+^ calcd for C_16_H_15_ClN_3_O: 300.0898, found: 300.0890; Anal. calcd for C_16_H_14_ClN_3_O: C 64.11, H 4.71, N 14.02, found: C 64.20, H 4.72, N 14.00.

**4-(2-(4-Chlorophenyl)-2-(1*H*-1,2,4-triazol-1-yl)ethyl)phenyl sulfamate (35)**: As method C using ClSO_2_NH_2_ (0.57 m, 6.0 mL), DMA (2.5 mL) and **34** (0.20 g, 0.67 mmol). Purification using a CombiFlash *R*_f_ (CH_2_Cl_2_/acetone) gave **35** as a white foam (0.22 g, 88 %): ^1^H NMR (270 mhz, [D_6_]DMSO): *δ*=3.45 (1 H, dd, *J*=14.0, 5.8 Hz, C*H*H), 3.66 (1 H, dd, *J*=14.0, 9.9 Hz, C*H*H), 5.97 (1 H, dd, *J*=9.9, 5.8 Hz, C*H*), 7.11 (2 H, AA′BB′, Ar*H*), 7.24 (2 H, AA′BB′, Ar*H*), 7.41–7.52 (4 H, m, Ar*H*), 7.94 (2 H, s, N*H*_2_), 7.99 (1 H, s, NC*H*N), 8.59 ppm (1 H, s, NC*H*N); ^13^C NMR (100 mhz, [D_6_]DMSO): *δ*=40.2 (CH_2_), 62.9 (CH_2_), 121.8 (2CH), 128.6 (2CH), 129.1 (2CH), 130.2 (2CH), 132.7 (C), 135.6 (C), 138.6 (C), 143.8 (CH), 148.8 (C), 151.7 ppm (CH); LC/MS (ESI+): *t*_R_=1.42 min, *m/z* (%): 379.0 (100) [*M*+H]^+^; HRMS-ESI: *m/z* [*M*+H]^+^ calcd for C_16_H_16_ClN_4_O_3_S: 379.0626, found: 379.0612.

**2-(3-Bromo-4-(triisopropylsilyloxy)phenyl)acetaldehyde (36)**: A solution of **25** (4.00 g, 10.7 mmol) in CH_2_Cl_2_ (6 mL) was added to a suspension of Dess–Martin reagent (5.46 g, 12.9 mmol) in CH_2_Cl_2_ (60 mL). After stirring for 1 h, Et_2_O (100 mL), satd aq NaHCO_3_ (100 mL) and satd aq Na_2_S_2_O_3_ (100 mL) were added and stirring continued for 10 min. The layers were separated, and the aqueous layer was extracted with Et_2_O (100 mL). The combined organic layers were washed with satd aq NaHCO_3_ (20 mL), H_2_O (20 mL) and brine (20 mL), then dried (MgSO_4_), filtered and concentrated in vacuo. Purification using a CombiFlash *R*_f_ (EtOAc/PE) gave **36** as a colourless oil (2.91 g, 73 %); ^1^H NMR (270 mhz, CDCl_3_): *δ*=1.08–1.37 (21 H, m, 3C*H*, 6C*H*_3_), 3.57 (2 H, d, *J*=2.2 Hz, C*H*_2_), 6.86 (1 H, d, *J*=8.5 Hz, Ar*H*), 6.97 (1 H, dd, *J*=8.3, 2.2 Hz, Ar*H*), 7.37 (1 H, d, *J*=2.2 Hz, Ar*H*), 9.70 ppm (1 H, t, *J*=2.2 Hz, C*H*O); ^13^C NMR (100 mhz, CDCl_3_): *δ*=13.0 (3CH), 18.1 (6CH_3_), 49.3 (CH_2_), 115.4 (C), 119.9 (CH), 125.4 (C), 129.5 (CH), 134.5 (CH), 152.5 (C), 199.1 ppm (CH).

**2-(3-Bromo-4-(triisopropylsilyloxy)phenyl)-1-(4-chlorophenyl)ethanol (37)**: 4-ClPhMgBr (1 m in Et_2_O, 15.5 mL) was added to a solution of **36** (2.85 g, 7.68 mmol) in Et_2_O (60 mL). After stirring for 2 h, the reaction was quenched by addition of H_2_O (30 mL) and 2 m HCl (30 mL). The product was extracted with Et_2_O (2×75 mL), and the combined organic layers were washed with brine (100 mL), then dried (MgSO_4_), filtered and concentrated in vacuo. Purification using a CombiFlash *R*_f_ (EtOAc/PE) gave **37** as white solid (2.66 g, 72 %): mp: 75–78 °C; ^1^H NMR (270 mhz, CDCl_3_): *δ*=1.06–1.36 (21 H, m, 3C*H*, 6C*H*_3_), 1.96 (1 H, d, *J*=3.0 Hz, O*H*), 2.86 (2 H, d, *J*=6.9 Hz, C*H*_2_), 4.82 (1 H, td, *J*=6.9, 3.0 Hz, C*H*), 6.77 (1 H, d, *J*=8.3 Hz, Ar*H*), 6.86 (1 H, dd, *J*=8.3, 2.2 Hz, Ar*H*), 7.18–7.35 ppm (5 H, m, Ar*H*); ^13^C NMR (100 mhz, CDCl_3_): *δ*=12.9 (3CH), 18.0 (6CH_3_), 44.8 (CH_2_), 74.6 (CH), 115.0 (C), 119.5 (CH), 127.3 (2CH), 128.5 (2CH), 129.3 (CH), 131.1 (C), 133.3 (C), 134.1 (CH), 141.9 (C), 151.8 ppm (C); LC/MS (ESI+): *t*_R_=2.54 min, *m/z* (%): 427.3 (100) [*M*+Na]^+^; HRMS-ESI: *m/z* [*M*+Na]^+^ calcd for C_23_H_33_ClNaO_2_Si: 427.1831, found: 427.1828.

**(2-Bromo-4-(2-chloro-2-(4-chlorophenyl)ethyl)phenoxy)triisopropylsilane (38)**: SOCl_2_ (2.44 g, 20.5 mmol) and DMF (5 drops) were added to a solution of **37** (2.48 g, 5.12 mmol) in CH_2_Cl_2_ (50 mL). After stirring for 2 h, the solvent was removed in vacuo, the residue was redissolved in CH_2_Cl_2_, and the process was repeated. Purification using a CombiFlash *R*_f_ (EtOAc/PE) gave **38** as a colourless oil (2.11 g, 82 %); ^1^H NMR (270 mhz, CDCl_3_): *δ*=1.06–1.35 (21 H, m, 3C*H*, 6C*H*_3_), 3.13 (1 H, dd, *J*=14.0, 7.2 Hz, C*H*H), 3.26 (1 H, dd, *J*=14.0, 7.2 Hz, C*H*H), 4.91 (1 H, t, *J*=7.2 Hz, C*H*), 6.73–6.75 (2 H, m, Ar*H*), 7.18–7.32 ppm (5 H, m, Ar*H*); ^13^C NMR (68 mhz, CDCl_3_): *δ*=13.0 (3CH), 18.1 (6CH_3_), 45.5 (CH_2_), 63.0 (CH), 112.5 (C), 119.5 (2CH), 128.6 (2CH), 128.7 (CH), 128.8 (C), 129.4 (CH), 130.8 (C), 134.2 (CH), 139.3 (C), 152.0 ppm (C).

**2-Bromo-4-(2-(4-chlorophenyl)-2-(1*H*-1,2,4-triazol-1-yl)ethyl)phenol (39)**: 1,2,4-Triazole (1.41 g, 20.43 mmol), K_2_CO_3_ (0.68 g, 4.93 mmol) and KI (0.067 g, 0.40 mmol) were sequentially added to a solution of **38** (2.04 g, 4.08 mmol) in acetone (80 mL). The reaction mixture was heated at 55 °C for 48 h then allowed to cool, and the solvent was removed in vacuo. The residue was redissolved in H_2_O/EtOAc (1:1, 160 mL), the layers were separated, and the aqueous layer extracted with EtOAc (80 mL). The combined organic layers were washed with brine (100 mL), then dried (MgSO_4_), filtered and concentrated in vacuo. Purification using a CombiFlash *R*_f_ (PE/EtOAc) and then (CH_2_Cl_2_/acetone) gave **39** as a white foam (0.25 g, 16 %): ^1^H NMR (270 mhz, [D_6_]DMSO): *δ*=3.27–3.34 (1 H, m, C*H*H), 3.49 (1 H, dd, *J*=13.8, 10.0 Hz, C*H*H), 5.86 (1 H, dd, *J*=9.9, 5.8 Hz, C*H*H), 6.73 (1 H, d, *J*=8.3 Hz, Ar*H*), 6.87 (1 H, dd, *J*=8.3, 1.9 Hz, Ar*H*), 7.32 (1 H, d, *J*=1.9 Hz, Ar*H*), 7.39–7.50 (4 H, m, Ar*H*), 7.98 (1 H, s, NC*H*N), 8.55 (1 H, s, NC*H*N), 10.06 ppm (1 H, s, O*H*); ^13^C NMR (100 mhz, [D_6_]DMSO): *δ*=38.9 (CH_2_), 63.1 (CH), 109.0 (C), 116.0 (CH), 128.5 (2CH), 129.1 (2CH), 129.2 (CH), 129.3 (CH), 132.7 (C), 133.1 (CH), 138.6 (C), 143.9 (CH), 151.6 (CH), 152.6 ppm (C); LC/MS (ESI+): *t*_R_=1.70 min, *m/z* (%): 378.0 (100) [*M*+H]^+^; HRMS-ESI: *m/z* [*M*+H]^+^ calcd for C_16_H_14_BrClN_3_O: 378.0003, found: 377.9993.

**2-Bromo-4-(2-(4-chlorophenyl)-2-(1*H*-1,2,4-triazol-1-yl)ethyl)phenyl sulfamate (40)**: As method C using ClSO_2_NH_2_ (0.57 m, 4.5 mL), DMA (1.5 mL) and a solution of **39** (0.15 g, 0.40 mmol) in DMA (1 mL). Purification using a CombiFlash *R*_f_ (CH_2_Cl_2_/acetone) gave **40** as a white foam (0.13 g, 71 %): ^1^H NMR (270 mhz, [D_6_]DMSO): *δ*=3.45 (1 H, dd, *J*=14.0, 5.8 Hz, C*H*H), 3.65 (1 H, dd, *J*=14.0, 10.2 Hz, C*H*H), 6.01 (1 H, dd, *J*=10.2, 5.8 Hz, C*H*), 7.20 (1 H, dd, *J*=8.5, 1.4 Hz, Ar*H*), 7.31 (1 H, d, *J*=8.5 Hz, Ar*H*), 7.42–7.52 (4 H, m, Ar*H*), 8.01 (1 H, s, NC*H*N), 8.59 (1 H, s, NC*H*N), 8.24 (2 H, s, N*H*_2_), 8.61 ppm (1 H, s, NC*H*N); ^13^C NMR (100 mhz, [D_6_]DMSO): *δ*=38.8 (CH_2_), 62.5 (CH_2_), 115.4 (C), 122.8 (CH), 128.6 (2CH), 129.1 (2CH), 129.5 (CH), 132.8 (C), 133.9 (CH), 137.3 (C), 138.4 (C), 143.9 (CH), 146.1 (C), 151.7 ppm (CH); LC/MS (ESI+): *t*_R_=1.42 min, *m/z* (%): 457.1 (100) [*M*+H]^+^; HRMS-ESI: *m/z* [*M*+H]^+^ calcd for C_16_H_15_BrClN_4_O_3_S: 456.9731, found: 456.9714.

**2-Bromo-4-((4-cyanophenyl)(1*H*-1,2,4-triazol-1-yl)methyl)phenyl dimethylsulfamate (42)**: *N,N*-Dimethylsulfamoyl chloride (0.60 g, 4.16 mmol) was added to a suspension of **41** (0.25 g, 0.70 mmol) in *N*,*N*-diisopropylethylamine (DIPEA; 5 mL). After heating at reflux for 1 h, the reaction mixture was allowed to cool, diluted with H_2_O and EtOAc, and then poured into 3 m HCl (100 mL). The layers were separated, and the organic layer was washed with H_2_O (50 mL) and satd aq NaHCO_3_ (50 mL), then dried (MgSO_4_), filtered and concentrated in vacuo. Purification using a Flashmaster II system (hexane/EtOAc) gave **42** as a light brown oil (0.11 g, 34 %): ^1^H NMR (270 mhz, CDCl_3_): *δ*=3.08 (6 H, s, 2C*H*_3_), 6.71 (1 H, s, C*H*), 7.13 (1 H, dd, *J*=8.4, 2.3 Hz, Ar*H*), 7.22–7.24 (2 H, m, Ar*H*), 7.41 (1 H, d, *J*=2.3 Hz, Ar*H*), 7.53 (1 H, d, *J*=8.4 Hz, Ar*H*), 7.69 (2 H, AA′BB′, Ar*H*), 8.04 (1 H, s, NC*H*N), 8.05 ppm (1 H, s, NC*H*N); ^13^C NMR (100 mhz, [D_6_]DMSO): *δ*=38.5 (2CH_3_), 63.4 (CH), 111.2 (C), 115.6 (C), 118.5 (C), 123.5 (CH), 128.9 (2CH), 129.4 (CH), 132.8 (2CH), 133.5 (CH), 138.2 (C), 143.6 (C), 145.1 (CH), 146.6 (C), 152.5 ppm (CH); LC/MS (ESI−): *t*_R_=4.23 min, *m/z* (%): 462.3 (100) [*M*−H]^−^; HRMS-FAB: *m/z* [*M*+H]^+^ calcd for C_18_H_17_BrN_5_O_3_S: 462.0230, found: 462.0220.

**1-Methyl-1*H*-benzo[*d*][1,2,3]triazole-6-carboxylic acid (44)**: NaNO_2_ (0.16 g, 2.32 mmol) dissolved in a small amount of H_2_O was added to a mixture of **43**[34] (0.26 g, 1.57 mmol) and 6 n HCl (2 mL) at 0 °C. After 5 min, the reaction was allowed to warm to RT and stirred for 3 h. H_2_O (20 mL) and EtOAc (20 mL) were added, the layers were separated, and the aqueous layer was extracted with EtOAc (20 mL). The combined organic layers were dried (MgSO_4_), filtered and concentrated in vacuo. Compound **44** was obtained as a brown solid and used without further purification (0.23 g, 82 %): ^1^H NMR (270 mhz, [D_6_]DMSO): *δ*=4.39 ppm (3 H, s, C*H*_3_), 7.92–7.97 (1 H, m, Ar*H* and C*H*), 8.15–8.09 (1 H, m, Ar*H*), 8.50 (1 H, s, Ar*H*); LC/MS (ESI+): *t*_R_=0.77 min, *m/z* (%): 175.5 (100) [*M*−H]^−^.

**Methyl 1-methyl-1*H*-benzo[*d*][1,2,3]triazole-6-carboxylate (45)**: SOCl_2_ (0.62 g, 4.52 mmol) added to a suspension of **44** (0.20 g, 1.13 mmol) in MeOH (15 mL), and the resulting mixture was heated at reflux overnight. The mixture was allowed to cool, and the solvent was removed in vacuo. Purification using a Flashmaster II (EtOAc/hexane) gave **45** as a cream solid (0.18 g, 83 %): ^1^H NMR (270 mhz, [D_6_]DMSO): *δ*=3.93 (3 H, s, OC*H*_3_), 4.40 (3 H, s, NC*H*_3_), 7.92–7.98 (1 H, m, Ar*H*), 8.13–8.18 (1 H, m, Ar*H*), 8.55 ppm (1 H, s, Ar*H*); LC/MS (ESI+): *t*_R_=0.87 min, *m/z* (%): 191.9 (100) [*M*+H]^+^, 159.7 (25).

**(1-Methyl-1*H*-benzo[*d*][1,2,3]triazole-6-yl)methanol (46)**: A solution of **45** (1.88 g, 9.84 mmol) in THF (25 mL) was added to a suspension of LiAlH_4_ (0.75 g, 19.7 mmol) in THF (60 mL). After stirring for 20 min, the reaction was quenched by addition of EtOAc (10 mL), H_2_O (10 mL) and 3 m HCl (50 mL). The product was extracted with EtOAc (2×75 mL), and the combined organics were washed with satd aq NaHCO_3_ (2×100 mL), then dried (MgSO_4_), filtered and concentrated in vacuo. Purification using a Flashmaster II (EtOAc/hexane) gave **46** as pale yellow crystals (1.07 g, 67 %): mp: 70–72 °C; ^1^H NMR (270 mhz, CDCl_3_): *δ*=4.11 (3 H, s, C*H*_3_), 4.82 (2 H, s, C*H*_2_), 4.87 (1 H, br s, O*H*), 7.21 (1 H, d, *J*=8.8 Hz, Ar*H*), 7.46 (1 H, s, Ar*H*), 7.76 ppm (1 H, d, *J*=8.8 Hz, Ar*H*); LC/MS (ESI+): *t*_R_=0.88 min, *m/z* (%): 163.9 (100) [*M*+H]^+^.

**1-Methyl-1*H*-benzo[*d*][1,2,3]triazole-6-carbaldehyde (47)**: Trichloroisocyanuric acid (0.81 g, 3.49 mmol) was added to a solution of **46** (0.54 g, 3.31 mmol) in CH_2_Cl_2_ (10 mL). The solution was cooled to 0 °C, TEMPO (0.006 g, 0.038 mmol) was added, the reaction mixture was stirred for 10 min and then filtered through Celite. The filtrate was washed with 20 % aq Na_2_CO_3_, 1 n HCl and brine, then dried (MgSO_4_), filtered and concentrated in vacuo to give **47** as a white solid used without further purification (0.50 g, 94 %): mp: 148.5–151 °C; ^1^H NMR (270 mhz, [D_6_]DMSO): *δ*=4.42 (3 H, s, C*H*_3_), 7.89 (1 H, dd, *J*=8.5, 1.4 Hz, Ar*H*), 8.21 (1 H, d, *J*=8.5 Hz, Ar*H*), 8.56 (1 H, d, *J*=1.4 Hz, Ar*H*), 10.19 ppm (1 H, s, C*H*O); LC/MS (ESI+): *t*_R_=0.90 min, *m/z* (%): 161.9 (100) [*M*+H]^+^.

**(4-(Benzyloxy)phenyl)(1-methyl-1*H*-benzo[*d*][1,2,3-triazole-6-yl)methanol (48)**: A solution of 4-benzyloxybromobenzene (1.35 g, 5.13 mmol) in THF (1 mL) was added in small portions to a suspension of Mg (0.11 g, 4.58 mmol) in THF (0.5 mL) containing a crystal of I_2_. Heating was used when required until the preparation of the Grignard reagent was complete. This reagent (4 equiv) were added to a suspension of **47** (0.050 g, 0.31 mmol) in THF (1 mL). After stirring overnight, H_2_O (5 mL) and HCl (1 m, 5 mL) were added, and the product was extracted with EtOAc (2×20 mL). The combined organic layers were washed with satd aq NaHCO_3_ (20 mL) and brine (20 mL), then dried (MgSO_4_), filtered and concentrated in vacuo. Purification using a Flashmaster II (EtOAc/hexane) gave **48** as a white crystalline solid (0.095 g, 89 %): mp: 131.5–133.5 °C; ^1^H NMR (270 mhz, [D_6_]DMSO): *δ*=4.30 (3 H, s, C*H*_3_), 5.06 (2 H, s, C*H*_2_), 5.85 (1 H, d, *J*=3.9 Hz, *CH*), 6.06 (1 H, d, *J*=3.9 Hz, OH), 6.93 (2 H, m, AA′BB′, Ar*H*), 7.25–7.45 (8 H, m, Ar*H*), 7.87–7.94 ppm (2 H, m, Ar*H*); ^13^C NMR (100 mhz, [D_6_]DMSO): *δ*=34.1 (CH_3_), 69.1 (CH_2_), 73.8 (CH), 106.8 (CH), 114.5 (2CH), 118.6 (CH), 123.2 (CH), 127.6 (2CH), 127.7 (2CH), 127.8 (CH), 128.4 (2CH), 133.4 (C), 137.2 (C), 137.7 (C), 144.3 (C), 145.6 (C), 157.3 ppm (C); LC/MS (ESI+): *t*_R_=1.64 min, *m/z* (%): 346.6 (100) [*M*+H]^+^; HRMS-ESI: *m/z* [*M*+H]^+^ calcd for C_21_H_20_N_3_O_2_: 346.1550, found: 346.1534.

**6-((4-(Benzyloxy)phenyl)(1*H*-1,2,4-triazol-1-yl)methyl)-1-methyl-1*H*-benzo[*d*][1,2,3]triazole (49)**: SOCl_2_ (0.1 mL) was added to a solution of **48** (0.090 g, 0.26 mmol) in CH_2_Cl_2_ (3 mL). After stirring for 2 h, the solvent was removed in vacuo and the residue was dissolved in acetone (5 mL). 1,2,4-Triazole (0.036 g, 0.52 mmol), KI (0.002 g, 0.012 mmol) and K_2_CO_3_ (0.11 g, 0.80 mmol) were added, and the reaction mixture was stirred at 60 °C overnight. H_2_O (10 mL) was added, and the product was extracted with EtOAc (2×10 mL). The combined organic layers were washed with H_2_O (20 mL), 1 m aq NaOH (20 mL) and brine (20 mL), then dried (MgSO_4_), filtered and concentrated in vacuo. Purification using a Flashmaster II (EtOAc/hexane) gave **49** as a yellow solid (0.061 g, 76 %): ^1^H NMR (400 mhz, [D_6_]DMSO): *δ*=4.25 (3 H, s, C*H*_3_), 5.09 (2 H, s, C*H*_2_), 7.02 (2 H, AA′BB′, Ar*H*), 7.20 (1 H, s, C*H*), 7.22–7.45 (8 H, m, Ar*H*), 7.65 (1 H, s, Ar*H*), 8.02 (1 H, d, *J*=9.0 Hz, Ar*H*), 8.09 (1 H, s, NC*H*N), 8.61 ppm (1 H, s, NC*H*N); ^13^C NMR (100 mhz, [D_6_]DMSO): *δ*=34.2 (CH_3_), 65.1 (CH), 69.3 (CH_2_), 109.6 (CH), 114.9 (2CH), 119.3 (CH), 124.3 (CH), 127.8 (2CH), 127.9 (CH), 128.5 (2CH), 129.6 (2CH), 130.8 (C), 133.3 (C), 136.9 (C), 138.8 (C), 144.5 (CH), 144.6 (C), 152.0 (CH), 158.2 ppm (C); LC/MS (ESI+): *t*_R_=1.62 min, *m/z* (%): 395.6 (100) [*M*]^+^; HRMS-ESI: *m/z* [*M*+H]^+^ calcd for C_23_H_21_N_6_O: 397.1771, found: 397.1756.

**4-((1-Methyl-1*H*-benzo[*d*][1,2,3]triazole-6-yl)(1*H*-1,2,4-triazol-1-yl)methyl)phenol (50)**: As method B using Pd/C (30 mg), **49** (0.28 g, 0.71 mmol) and THF/MeOH (1:1, 20 mL). Purification using a Flashmaster II (CH_2_Cl_2_/MeOH) gave **50** as a white solid (0.21 g, 97 %): ^1^H NMR (270 mhz, [D_6_]DMSO): *δ*=4.25 (3 H, s, C*H*_3_), 6.75 (2 H, AA′BB′, Ar*H*), 7.09 (2 H, AA′BB′, Ar*H*), 7.16 (1 H, s, C*H*), 7.27 (1 H, d, *J*=8.5 Hz, Ar*H*), 7.61 (1 H, s, Ar*H*), 8.02 (1 H, d, *J*=8.5 Hz, Ar*H*), 8.08 (1 H, s, NC*H*N), 8.56 (1 H, s, NC*H*N), 9.63 ppm (1 H, s, O*H*); ^13^C NMR (100 mhz, [D_6_]DMSO): *δ*=34.2 (CH_3_), 65.4 (CH), 109.5 (CH), 115.4 (2CH), 119.2 (CH), 124.3 (CH), 128.8 (C), 129.6 (2CH), 133.3 (C), 139.0 (C), 144.3 (CH), 144.6 (C), 152.0 (CH), 157.3 ppm (C); LC/MS (ESI−): *t*_R_=1.24 min, *m/z* (%): 305.2 (100) [*M*−H]^−^; HRMS-ESI: *m/z* [*M*+H]^+^ calcd for C_16_H_15_N_6_O: 307.1302, found: 307.1296.

**4-((1-Methyl-1*H*-benzo[*d*][1,2,3]triazole-6-yl)(1*H*-1,2,4-triazol-1-yl)methyl)phenyl sulfamate (51)**: As method C using ClSO_2_NH_2_ (0.45 m, 5.0 mL), DMA (2.5 mL) and **50** (0.11 g, 0.36 mmol). Purification using a Flashmaster II (CH_2_Cl_2_/acetone) gave **51** as a white solid (0.12 g, 84 %): ^1^H NMR (270 mhz, [D_6_]DMSO): *δ*=4.27 (3 H, s, C*H*_3_), 7.26–7.39 (6 H, m, Ar*H* and C*H*), 7.72 (1 H, s, Ar*H*), 8.00 (2 H, s, N*H*_2_), 8.05 (1 H, d, *J*=8.8 Hz, Ar*H*), 8.13 (1 H, s, NC*H*N), 8.66 ppm (1 H, s, NC*H*N); ^13^C NMR (100 mhz, [D_6_]DMSO): *δ*=34.3 (CH_3_), 64.9 (CH), 110.0 (CH), 119.4 (CH), 122.5 (2CH), 124.4 (CH), 129.7 (2CH), 133.4 (C), 137.0 (C), 138.1 (C), 144.7 (C, CH), 149.8 (C), 152.2 ppm (CH); LC/MS (ESI−): *t*_R_=1.23 min, *m/z* (%): 384.4 (100) [*M*−H]^−^; HRMS-ESI: *m/z* [*M*+H]^+^ calcd for C_16_H_16_N_7_O_3_S: 386.1030, found: 386.1013.

**(*R*)-4-(2-(3-Bromo-4-hydroxyphenyl)-1-(1*H*-1,2,4-triazol-1-yl)ethyl)benzonitrile (17 a)**: Separation of **17** on a Chiralpak AD-H (250×20 mm) semi-prep column as described in the text gave **17 a**: *t*_1_=7.92 min; 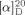
=−18.7° (*c*=0.89 in EtOH).

**(*S*)-4-(2-(3-Bromo-4-hydroxyphenyl)-1-(1*H*-1,2,4-triazol-1-yl)ethyl)benzonitrile (17 b)**: Separation of **17** on a Chiralpak AD-H (250×20 mm) semi-prep column as described in the text gave **17 b**: *t*_2_=15.96 min; 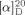
=+25.1° (*c*=0.90 in EtOH).

**(*R*)-2-Bromo-4-(2-(4-cyanophenyl)-2-(1*H*-1,2,4-triazol-1-yl)ethyl)phenyl sulfamate (18 a)**: Prepared from **17 a** as described for (±)-**18**: 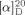
=−15.9° (*c*=4.1 in EtOH).

**(*S*)-2-Bromo-4-(2-(4-cyanophenyl)-2-(1*H*-1,2,4-triazol-1-yl)ethyl)phenyl sulfamate (18 b)**: Prepared from **17 b** as described for (±)-**18**: 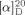
=+16.5° (*c*=3.1 in EtOH).

## References

[1] Brueggemeier RW, Hackett JC, Diaz-Cruz ES (2005). Endocr. Rev..

[2] Brodie A (2002). Trends Endocrinol. Metab..

[3] Recanatini M, Cavalli A, Valenti P (2002). Med. Res. Rev..

[4] Dixon JM (2006). Expert Opin. Pharmacother..

[5] Howell A, Cuzick J, Baum M, Buzdar A, Dowsett M, Forbes JF, Hoctin-Boes G, Houghton I, Locker GY, Tobias JS (2005). Lancet.

[6] The Breast International Group 1–98 Collaborative (2005). N. Engl. J. Med..

[7] Geisler J, Helle H, Ekse D, Duong NK, Evans DB, Nordbo Y, Aas T, Lonning PE (2008). Clin. Cancer Res..

[8] Monnier A (2006). Expert Rev. Anticancer Ther..

[9] Reed MJ, Purohit A, Woo LWL, Newman SP, Potter BVL (2005). Endocr. Rev..

[10] Santner SJ, Feil PD, Santen RJ (1984). J. Clin. Endocrinol. Metab..

[11] Poulin R, Labrie F (1986). Cancer Res..

[12] Dauvois S, Labrie F (1989). Breast Cancer Res. Treat..

[13] Howarth NM, Purohit A, Reed MJ, Potter BVL (1994). J. Med. Chem..

[14] Woo LWL, Howarth NM, Purohit A, Hejaz HAM, Reed MJ, Potter BVL (1998). J. Med. Chem..

[15] Stanway SJ, Purohit A, Woo LWL, Sufi S, Vigushin D, Ward R, Wilson RH, Stanczyk FZ, Dobbs N, Kulinskaya E, Elliott M, Potter BVL, Reed MJ, Coombes RC (2006). Clin. Cancer Res..

[16] Morphy R, Rankovic Z (2005). J. Med. Chem..

[17] Espinoza-Fonseca LM (2006). Bioorg. Med. Chem..

[18] Meunier B (2008). Acc. Chem. Res..

[19] Wood PM, Woo LWL, Humphreys A, Chander SK, Purohit A, Reed MJ, Potter BVL (2005). J. Steroid Biochem. Mol. Biol..

[20] Wood PM, Woo LWL, Labrosse JR, Trusselle MN, Abbate S, Longhi G, Castiglioni E, Lebon F, Purohit A, Reed MJ, Potter BVL (2008). J. Med. Chem..

[21] Woo LWL, Sutcliffe OB, Bubert C, Grasso A, Chander SK, Purohit A, Reed MJ, Potter BVL (2003). J. Med. Chem..

[22] Woo LWL, Bubert C, Sutcliffe OB, Smith A, Chander SK, Mahon MF, Purohit A, Reed MJ, Potter BVL (2007). J. Med. Chem..

[23] Bubert C, Woo LWL, Sutcliffe OB, Mahon MF, Chander SK, Purohit A, Reed MJ, Potter BVL (2008). ChemMedChem.

[24] Wood PM, Woo LWL, Labrosse JR, Thomas MP, Mahon MF, Chander SK, Purohit A, Reed MJ, Potter BVL (2010). ChemMedChem.

[25] Jackson T, Woo LWL, Trusselle MN, Chander SK, Purohit A, Reed MJ, Potter BVL (2007). Org. Biomol. Chem..

[26] Woo LWL, Jackson T, Putey A, Cozier G, Leonard P, Acharya KR, Chander SK, Purohit A, Reed MJ, Potter BVL (2010). J. Med. Chem..

[27] Abbate S, Longhi G, Castiglioni E, Lebon F, Wood PM, Woo LWL, Potter BVL (2009). Chirality.

[28] Bowman RM, Steele RE, Browne L (1990).

[29] Woo LWL, Purohit A, Malini B, Reed MJ, Potter BVL (2000). Chem. Biol..

[30] Ohshima T, Gnanadesikan V, Shibuguchi T, Fukuta Y, Nemoto T, Shibasaki M (2003). J. Am. Chem. Soc..

[31] Okada M, Iwashita S, Koizumi N (2000). Tetrahedron Lett..

[32] Karjalainen A, Kalapudas A, Sodervall M, Pelkonen O, Lammintausta R (2000). Eur. J. Pharm. Sci..

[33] Avery MA, Muraleedharan KM, Desai PV, Bandyopadhyaya AK, Furtado MM, Tekwani BL (2003). J. Med. Chem..

[34] Dener JM, O’Bryan C, Yee R, Shelton EJ, Sperandio D, Mahajan T, Palmer JT, Spencer JR, Tong ZW (2006). Tetrahedron Lett..

[35] Raeymaekers AHM, Freyne EJE, Van Gelder JLH, Venet MG (1990).

[36] De Luca L, Giacomelli G, Porcheddu A (2001). Org. Lett..

[37] Vanden Bossche H, Willemsens G, Roels I, Bellens D, Moereels H, Coene MC, Lejeune L, Lauwers W, Janssen PAJ (1990). Biochem. Pharmacol..

[38] Jones CD, Winter MA, Hirsch KS, Stamm N, Taylor HM, Holden HE, Davenport JD, Vonkrumkalns EV, Suhr RG (1990). J. Med. Chem..

[39] Gobbi S, Cavalli A, Negri M, Schewe KE, Belluti F, Piazzi L, Hartmann RW, Recanatini M, Bisi A (2007). J. Med. Chem..

[40] Recanatini M, Bisi A, Cavalli A, Belluti F, Gobbi S, Rampa A, Valenti P, Palzer M, Palusczak A, Hartmann RW (2001). J. Med. Chem..

[41] Kovacs A, Varga Z (2006). Coord. Chem. Rev..

[42] Woo LWL, Purohit A, Reed MJ, Potter BVL (1997). Bioorg. Med. Chem. Lett..

[43] Ho YT, Purohit A, Vicker N, Newman SP, Robinson JJ, Leese MP, Ganeshapillai D, Woo LWL, Potter BVL, Reed MJ (2003). Biochem. Biophys. Res. Commun..

[44] Purohit A, Chander SK, Woo LWL, Parsons MFC, Jhalli R, Potter BVL, Reed MJ (2008). Anticancer Res..

[45] Goss PE (1998). Breast Cancer Res. Treat..

[46] Chander SK, Purohit A, Woo LWL, Potter BVL, Reed MJ (2004). Biochem. Biophys. Res. Commun..

[47] Furet P, Batzl C, Bhatnagar A, Francotte E, Rihs G, Lang M (1993). J. Med. Chem..

[49] Cavalli A, Bisi A, Bertucci C, Rosini C, Paluszcak A, Gobbi S, Giorgio E, Rampa A, Belluti F, Piazzi L, Valenti P, Hartmann RW, Recanatini M (2005). J. Med. Chem..

[50] Ghosh D, Griswold J, Erman M, Pangborn W (2009). Nature.

[51] Jones G, Willett P, Glen RC, Leach AR, Taylor R (1997). J. Mol. Biol..

[52] Hernandez-Guzman FG, Higashiyama T, Pangborn W, Osawa Y, Ghosh D (2003). J. Biol. Chem..

[53] Appel R, Berger G (1958). Chem. Ber..

[54] Woo LWL, Lightowler M, Purohit A, Reed MJ, Potter BVL (1996). J. Steroid Biochem. Mol. Biol..

