# Supplementary material for: Aromatase and Dual Aromatase-Steroid Sulfatase Inhibitors from the Letrozole and Vorozole Templates
Source: ChemMedChem. 2011 May 23;6(8):1423–38. doi: 10.1002/cmdc.201100145 (PMC3170879; doi:10.1002/cmdc.201100145)

CHEMMEDCHEM
Chemistry & Drug Discovery

Supporting Information

© Copyright Wiley-VCH Verlag GmbH & Co. KGaA, 69451 Weinheim, 2011

Aromatase and Dual Aromatase-Steroid Sulfatase Inhibitors from the Letrozole and Vorozole Templates

Paul M. Wood,[a] L. W. Lawrence Woo,[a] Mark P. Thomas,[a] Mary F. Mahon,[b] Atul Purohit,[c] and Barry V. L. Potter*[a]

cmdc_201100145_sm_miscellaneous_information.pdf

Supporting Information

Contents:

HPLC data (page 1)

Chiral HPLC (page 2)

HPLC Data

Compound	HPLC Purity (%)	R_t (min)	Solvent System
**10**	96.8	1.66	90:10 $CH_3CN:H_2O$
**11**	>99	1.72	90:10 $CH_3CN:H_2O$
**14**	>99	1.29	90:10 $CH_3CN:H_2O$
**17**	>99	1.38	90:10 $CH_3CN:H_2O$
**18**	>99	2.11	70:30 $CH_3CN:H_2O$
**21**	>99	1.36	90:10 $CH_3CN:H_2O$
**22**	>99	1.33	90:10 $CH_3CN:H_2O$
**28**	>99	1.41	90:10 $CH_3CN:H_2O$
**29**	>99	1.35	90:10 $CH_3CN:H_2O$
**35**	>99	1.43	90:10 $CH_3CN:H_2O$
**39**	>99	1.60	90:10 $CH_3CN:H_2O$
**40**	>99	1.46	90:10 $CH_3CN:H_2O$
**42**	97.3	1.37	90:10 $CH_3CN:H_2O$
**50**	97.3	1.39	90:10 $CH_3CN:H_2O$
**51**	>99	1.37	90:10 $CH_3CN:H_2O$

Chiral HPLC data

Analytical chiral HPLC was performed with a Chiralpak® AD-H column (250x4.6mm, 5µm) with methanol as the mobile phase, a flow rate of 1.2 mL/min and a PDA detector. Semi-preparative HPLC was performed with a Waters 2525 binary gradient module and a Chiralpak® AD-H (250 x 20 mm) semi-prep column with MeOH as the mobile phase at a flow rate of 10 mL/min, injecting 1.5-2.0 mL of a 20 mg/mL solution and a run time of 25 minutes. Example HPLC traces obtained following analysis of batches of **17a** and **17b** following separation by prep-HPLC are shown below.

For **17a**, t_r = 3.80 min.

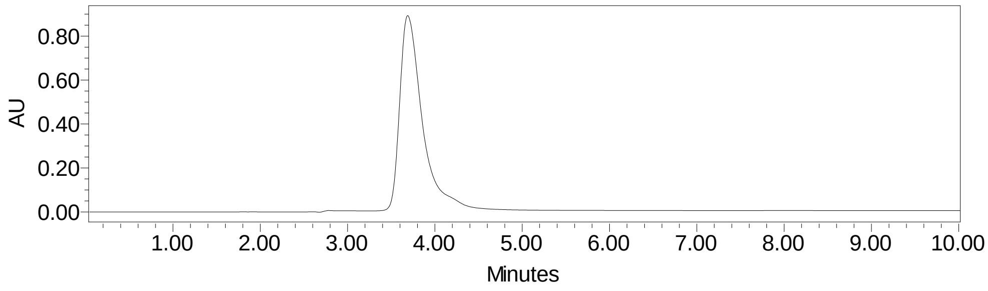

For **17b**, t_r = 8.20 min.

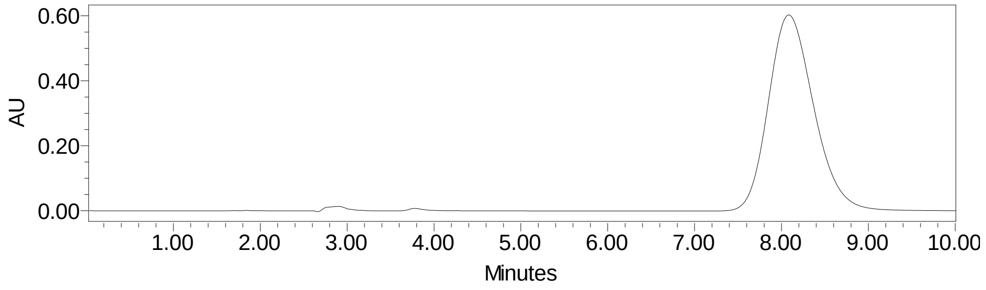

Supplement: Supplementary file 1 [file cmdc0006-1423-SD1.pdf]
